# Insights into the origin of the invasive populations of *Trioza erytreae* in Europe using microsatellite markers and mtDNA barcoding approaches

**DOI:** 10.1038/s41598-021-97824-0

**Published:** 2021-09-20

**Authors:** Omar Ruíz-Rivero, Andrés Garcia-Lor, Borja Rojas-Panadero, José Carlos Franco, Fathiya M. Khamis, Kerstin Kruger, Dina Cifuentes, Pablo Bielza, Alejandro Tena, Alberto Urbaneja, Meritxell Pérez-Hedo

**Affiliations:** 1 Instituto Valenciano de Investigaciones Agrarias (IVIA), Centro de Protección Vegetal y Biotecnología, CV-315 Km 10.7, 46113 Moncada, Valencia, Spain; 2 Centro de Citricultura y Producción Vegetal, Instituto Valenciano de Investigaciones Agrarias (IVIA), CV-315 Km 10.7, 46113 Moncada, Valencia, Spain; 3 Instituto de Biotecnología Vegetal, Universidad Politécnica de Cartagena (UPCT), Cartagena, Spain; 4 Centro de Estudos Florestais, Instituto Superior de Agronomia, Universidade de Lisboa, Tapada da Ajuda, 1349-017 Lisbon, Portugal; 5 International Centre of Insect Physiology and Ecology (ICIPE), P.O. Box 30772-00100, Nairobi, Kenya; 6 Department of Zoology and Entomology, University of Pretoria, Private Bag X20, Pretoria, 0028 South Africa

**Keywords:** Entomology, Ecological genetics, Genetic variation

## Abstract

The African citrus psyllid *Trioza erytreae* is one of the major threats to citrus industry as the vector of the incurable disease known as huanglongbing (HLB) or citrus greening. The psyllid invaded the northwest of the Iberian Peninsula 6 years ago. The invasion alarmed citrus growers in the Mediterranean basin, the largest citrus producing area in Europe, which is still free of HLB. Before our study, no research had been carried out on the genetic diversity of *T. erytreae* populations that have invaded the Iberian Peninsula and the archipelagos of the Macaronesia (Madeira and the Canary Islands). In this study, combining microsatellites markers and mtDNA barcoding analysis, we characterize the genetic diversity, structure and maternal relationship of these new invasive populations of *T. erytreae* and those from Africa. Our results suggest that the outbreaks of *T. erytreae* in the Iberian Peninsula may have derived from the Canary Islands. The populations of *T. erytreae* that invaded Macaronesia and the Iberian Peninsula are likely to have originated from southern Africa. We anticipate our results to be a starting point for tracking the spread of this invasive pest outside of Africa and to be important for optimizing contingency and eradication plans in newly invaded and free areas.

## Introduction

The African citrus psyllid *Trioza erytreae* (Del Guercio) (Hemiptera: Triozidae) is an invasive pest that has become one of most severe threats to the Mediterranean citrus industry^1–3^. Along with the Asian citrus psyllid *Diaphorina citri* Kuwayama (Hemiptera: Psyllidae), *T. erytreae* is a vector of the phloem-restricted Gram-negative bacteria *Candidatus* Liberibacter species that occur in citrus. These bacteria are the causative agents of citrus greening disease, an incurable and most devastating disease that affect citrus, also known as huanglongbing (*yellow dragon disease*) or simply by its acronym HLB^4,5^. Spread of HLB by *T. erytreae* had a devastating impact on citrus production in the cooler highland regions of Kenya and Tanzania, where it caused losses of 25–100%^6^. In Florida, the largest orange growing area in the United States of America, the spread of HLB by *D. citri* caused citrus production decline by 74%, resulting in losses of about USD 4554 million^7,8^. Besides their role as HLB vectors, the nymphs of both psyllid species excrete large amount of honeydew that facilitates the growth of sooty moulds on infested trees^2,9^.

*Trioza erytreae* was first reported in 1897 in the South African regions of the Eastern Cape and Stellenbosch^10^, but it was not until 1918 that Del Guercio first described the African citrus psyllid from samples collected on lemon trees in Eritrea (Ethiopia)^11^. *Trioza erytreae* has been reported in Sub-Saharan Africa, e.g. Kenya (Eastern Africa), South Africa (Southern Africa), the South Atlantic island of Saint Helena, in the Indian Ocean islands of Madagascar, in addition to Sudan (in north-eastern Africa)^4^. *Trioza erytreae* has also been found in the Arabian Peninsula, where it was likely introduced into Yemen from Ethiopia^12^. In 1994, *T. erytreae* was found in the archipelagos of the Macaronesia, in the North Atlantic Ocean. It was first reported in Madeira^13^ and 8 years later in the Canary Islands^14^. *Trioza erytreae* has become adventive in a wide range of different geographic locations, altitudinal tiers (from sea level to 1300 m above sea level), different environmental conditions (from hot with frequent rains to cool and moist, or even hot and dry weathers)^2,15^, and to a wide range of host species mainly within Rutaceae^16^.

In mainland Europe, *T. erytreae* was recorded for first time in the summer of 2014, specifically in the northwest of the Iberian Peninsula, in Pontevedra (Spain), and Porto (Portugal)^17^. Since then, its distributional range in the Iberian Peninsula has expanded rapidly to the point of becoming a serious threat to the entire citrus industry based in the Mediterranean basin^2^. Between 2017 and 2018, the psyllid moved more than 200 km southwards along the west coast of Portugal, from Figueira da Foz (Coimbra) in the north to Pontes (Setúbal) in the south. Currently, the distribution of *T. erytreae* in the west coast of the Iberian Peninsula covers a straight uninterrupted line of 600 km from Cedeira (A Coruña, Spain) to Setúbal (Portugal)^18^. At the present, no further southerly movement of the psyllid has been detected towards the closet major European citrus growing areas (the Algarve in Portugal and Huelva in Spain, 170 and 200 km away, respectively). In Spain, the distribution of *T. erytreae* has expanded in a north-eastern direction throughout to the Cantabrian coast, and specific outbreaks have been reported recently in Asturias, Cantabria and the Basque Country^19^. So far it is still unknown how *T. erytreae* reached the Iberian Peninsula.

Although the HLB has not been detected in the mainland of Spain and Portugal, the alarm of citrus growers in the Mediterranean basin, particularly in Spain—the largest citrus fruit producer in Europe—is increasing due to the rapid spread of *T. erytreae*. The wide distribution of *T. erytreae* makes any contingency measure a challenging effort^20^. It has been demonstrated that the parasitoid wasp *Tamarixia dryi* (Waterston) (Hymenoptera: Eulophidae) is a highly specific parasitoid of *T. erytreae*^21,22^, and its use in classical biological control programme has decreased the African citrus psyllid populations in the Canary Islands drastically until they have almost totally disappeared^23^. A *T. dryi*-assisted biological control program against *T. erytreae* was also carried out successfully in the Indian Ocean island of Réunion ^24^ and Mauritius^25^.

Knowledge of the geographical origin of invasive insects is essential for developing effective contingency measures against these threatening pests. In this regard, it has been demonstrated that information on genetic variation among populations can be used to assess this question^26^. The first report on the intraspecific genetic diversity of the African citrus psyllid, suggested that *T. erytreae* recorded in Europe most likely originated from South Africa, although the possibility of a Kenyan origin could not be ruled out based solely on the use of the *COI* mitochondrial genetic marker (mtDNA barcoding)^27^.

Apart from mtDNA barcoding, nuclear markers such as the microsatellites (SSRs: Simple Sequence Repeats) have been used widely, because of their extensive genome distribution and high level of polymorphism, to study the genetic diversity and phylogeography of several crop pest insects such the tomato leaf miner *Tuta* absoluta (Meyrick)^28^ (Lepidoptera: Gelechiidae); the beet armyworm *Spodoptera exigua* (Hübner)^29^ (Lepidoptera: Noctuidae); the sugar cane aphid *Melanaphis sacchari* (Zehntner)^30^ (Hemiptera: Aphididae); the cotton mealybug *Phenacoccus solenopsis* (Tinsley)^31^ (Hemiptera: Pseudococcidae); the whitefly *Bemisia tabaci* (Gennadius)^3232^ (Hemiptera: Aleyrodidae); as well as the Asian citrus psyllid *D. citri*^33,34^. The main objective of the present work is to gain insights in the genetic structure of the *T. erytreae* populations, as very little is known about their genetic traits. The inference of the origin and colonisation routes of the *T. erytreae* invasive populations in Europe is critical for the design and implementation of accurate management strategies against this devastating pest. We used nuclear SSR markers and mtDNA barcoding to analyse the genetic diversity and phylogeny of *T. erytreae* populations collected from different locations in Galicia and Canary Islands, Madeira, Portugal mainland, and Africa (Kenya and South Africa).

## Results

### Genome-wide characterisation of SSRs

**Figure 1:**
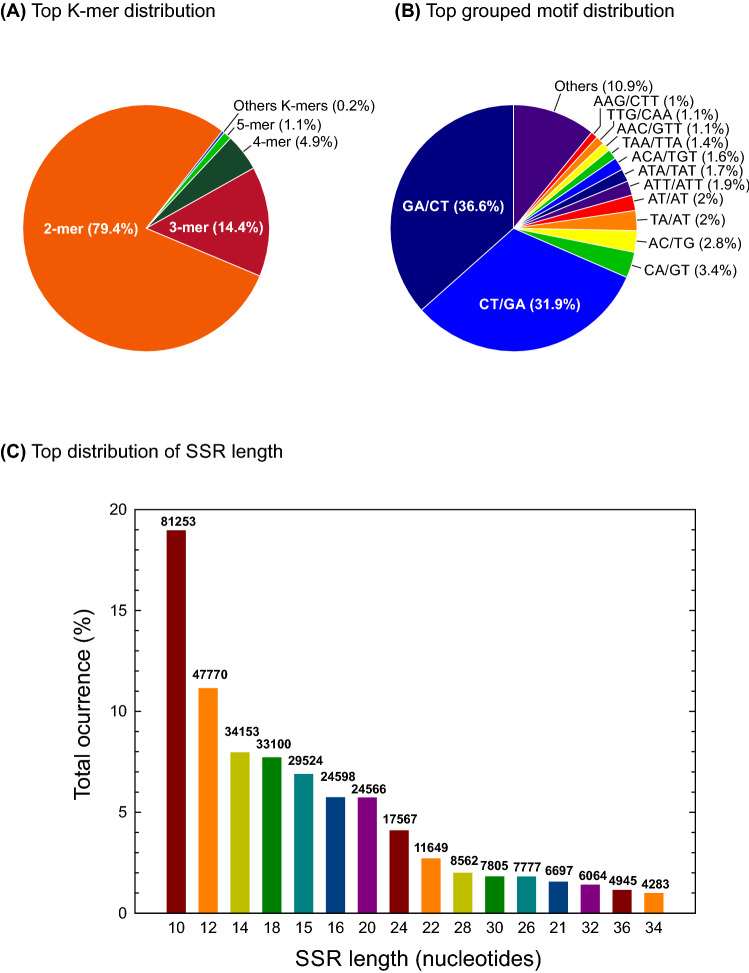
Frequency distribution of different classes of SSR repeat units in the *Trioza erytreae* genome. **(A)** Frequency of motif types by unit length (K-mers). **(B)** Frequency of grouped repeated motifs by nucleotide composition. **(C)** Length distribution of SSRs (total number of each type of SSR length is shown in the top of the bars).

We identified and mapped a total of 428,342 microsatellites across the 47,828 scaffolds of the unpublished genome sequence draft of *T. erytreae* using the GMATA software^35^. The SSRs frequency was estimated at 765.6 SSRs/Mb, which means 1 SSR for every 1.09 Kb. In silico identified SSRs were distributed among ten types of in tandem repeated motifs (from di- to deca-nucleotides). Analysis of SSR distribution revealed that the di-nucleotide motifs (340,227) were the most abundant SSRs, with a frequency of 79.4%. Both tetra- (20,902) and tri- (61,839) nucleotide repeats comprised about 5–15% (Fig. 1A; Supplementary Data 1). The remaining motifs, from hepta- to deca-nucleotides, comprised less than 1.5% of total SSRs identified in this study (Fig. 1A). Considering the unknown orientation of DNA strands in the Tery6 draft genome sequence of *T. erytreae*, a further SSRs characterization was carried out grouping the repeat motifs into pairs of complementary sequences. According to this, GA/TC (36.6%) and CT/AG (31.9%) are the most frequent motif pairs, with a total frequency of 68.5% (Fig. 1B). Grouped motif pairs GC/GC (0.05%) and CG/CG (~ 0.02%) were the least abundant di-nucleotide motifs. In decrease order, the most abundant tri-nucleotide motif pairs were ATT/AAT, ATA/TAT, ACA/TGT, TAA/TTA, AAC/GTT, TTG/CAA, and AAG/CTT, which encompassed 9.8% of all identified grouped motif pairs. Occurrence frequency of the remaining grouped motifs, including the rest of tri- and those from tetra- to deca-nucleotides (552 all together), was less than 11% of all motif pairs (Fig. 1B). Our data analysis reveals that SSR markers of 10 bp were most frequent, accounting for about 10% all SSR markers identified in this study. The overall trend of SSR length distribution in the *T. erytreae* genome is that the frequency of occurrence of SSRs gradually decreases as their length increases (Fig. 1C).

### SSR markers development for *T. erytreae*

Fifteen SSRs chosen from those repeated motifs identified in silico in this study (Table 1) were used as potential markers to investigate the genetic diversity, structure and phylogeography of *T. erytreae* individuals from populations in mainland Europe and the archipelagos of the Macaronesia. Scaffolds Tery6_s00034 (274,710 bp), Tery6_s02825 (48,689 bp) and Tery6_s07841 (26739 bp) were randomly selected based on their sequence length (long, medium, and short scaffolds, respectively). SSRs were selected on the base of their type of repeat motif (di, tri-, tetra- and penta-nucleotides), nucleotide composition and length (number of in tandem repeated motifs) (Table 1; Supplementary Data 2). For the scaffold Tery6_s00034, 11 SSR loci were chosen from the total of 106 SSRs identified in silico, three for Tery6_s07841 and one for Tery6_s02825. Selected scaffolds were further investigated to know whether SSR loci mapped into coding or non-coding regions (inter-genic or intron sequences). Although gene annotation of the *T. erytreae* genome draft is not yet completed, it was possible to get this information for most of the selected SSR loci (*data not shown*). The scaffolds Tery6_s00034, Tery06, − 07, − 13 and − 14 were found in inter-genic regions, while Tery08, − 12 and − 15 were mapped into introns. For Tery05, − 9, − 10 and − 11 was not possible to establish whether they targeted coding or non-coding regions. SSR loci Tery01, − 02 and − 03 were found in intron regions in the scaffold Tery6_s07841, and SSR locus Tery04 in an inter-genic region in the sequence corresponding to the scaffold Tery6_s02825. For amplification of SSR loci, specific PCR primers were designed on the sequence flanking the in tandem repeated motifs. Blast of the different amplicons against the *T. erytreae* draft genome sequence showed that PCR primers would result in the specific amplification of their specific SSR locus. Experimental validation of PCR primers was carried out on a testing panel of individuals collected in different locations in the Canary Islands and South Africa. Primers pairs for SSR loci Tery04, − 05, − 06, − 08, − 09, − 10, − 11, − 12, − 13 and − 15 yielded DNA fragments of the expected size and were chosen for carry on further population genetic analysis. These loci contain eight di-nucleotides (AC, AG, GA, CA, GT, TC, TA and TG), one tri-nucleotide (TGA), and three tetra-nucleotides (CATA, CTAC and TACC), which arranged in microsatellites of different length (from 5 to 30 in tandem repeated motifs) (Table 1). Five SSR loci (Tery01, − 02, − 03, − 07 and − 14) were not amplified efficiently and the corresponding primer pairs were discarded for further analysis.

**Table 1:** SSR loci developed in *Trioza erytreae.*

SSR locus	Tery6 scaffold	Primer sequence (5′ → 3′)*	PCR product (bp)	Repeat units
Tery01	07841	FW: CCAACTCCTGGGCTCAAACCCT	332	(ATTCT)_6_N_90_(CTATT)_6_
		RV: CAGCAGAGAAATGTGAGACTGGTCTGA		
Tery02	s07841	FW: CAATGAGATGTTGCACATGCAGGAT	225	(ACC)_12_
		RV: CGTGTCAGCATCTTTCTCCACAGGT		
Tery03	s07841	FW: AGAGCCACTAATGAGATGTTGCACATG	234	(ACC)_12_
		RV: CCGTGTCAGCATCTTTCTCCACAG		
Tery04	s02825	FW: ATCCATTTTGCTTCCCTGCTCG	287	(AC)_27_
		RV: TGTTCAGTGTTCAACCCAACTTTATCGT		
Tery05	s00034	FW: GACAATGTTAGGGTTAAGGATGAATGATGAT	252	(CTAC)_5_N_5_(TACC)_5_
		RV: GGGTTCGGGCAAGGATTGTAGG		
Tery06	s00034	FW: CGACCGTCAGACTGTTAATATCCATCAG	294	(CATA)_15_/(AT)_5_
		RV: GCAATCATCTGAAATAACTTTCCATTTTTGTAC		
Tery07	s00034	FW: ATTGGATCCCTGAGGCATGGC	287	(TG)_5_N_3_8(TG)5
		RV: CTCAGGTGGTGTGCATGATTTGGTA		
Tery08	s00034	FW: TTCAGTCTTCCGAACAAGGGCAGT	318	(AG)_5_N_17_(GA)_18_N_2_(GA)_14_
		RV: GGACGGAGAAGGAACTAAGTTAATTGAGTT		
Tery09	s00034	FW: TTATCTTTCCCACTTTATCTGATAATTCCTCG	270	(TA)_14_(TG)_20_
		RV: TCCGAACTCGACCGACAGATCG		
Tery10	s00034	FW: GAGAGGTAAACCTAAATTATGCTGCTCCA	359	(AG)_11_AT(AG)_22_
		RV: CGTCTTTGATTCACTCAAGTAGCCCAT		
Tery11	s00034	FW: TCTTTCAAATTAAGTACCATTTCATTCCCTCTA	319	(TC)_30_
		RV. TCTGTCTAAAACAAGCCGCTGCG		
Tery12	s00034	FW: AATTGAAGGGAAGGAGGAGAGAATAGGTAT	278	(TGA)_7_
		RV: GCATTGGATTCACCCTGGCGT		
Tery13	s00034	FW: CGACCGTCAGACTGTTAATATCCATCAG	267	(CA)_6_
		RV: CCCTATTTGTTGACTATGGATACTTGACTGC		
Tery14	s00034	FW: ATTTTCGACTTGAAATAAGAAGAGCTATCCA	277	(TG)_6_TC(TG)_3_TC(TG)_5_
		RV: AGACATGAAACCTAGCCTATAACCACCG		
Tery15	s00034	FW: ACTTATTTGGCGTCGTCGGCG	443	(TA)_9_
		RV: TCCGAACTCGACCGACAGATCG		

*FW forward; *RV* reverse.

The individuals of *T. erytreae* collected in different geographical locations in the west coast of mainland Spain and Portugal, the Canary Islands and Madeira, as well as in South Africa and Kenya (Table 2), were analysed using the 10 selected SSR markers designed in this study. The scored allelic data for each SSR marker is summarised in the Table 3. The analysis showed that all SSR markers were polymorphic. Seventy alleles were detected over the ten selected SSR loci, and the average number of alleles per locus (*N*_a_) was seven. SSR markers Tery08 and Tery11 had the highest number of alleles (12 and 20 alleles respectively), whereas Tery13 had the lowest (only two alleles). The expected (*H*_e_) and observed (*H*_o_) heterozygosity per locus in the entire population ranged from 0.20 to 0.77 and from 0.03 to 0.84, respectively. SSRs Tery11 and Tery08 displayed the highest diversity (*H*_e_ of 0.77 and 0.72, respectively), and Tery09 and Tery13 (*H*_e_ of 0.20 and 0.22, respectively) were the least informative markers. Most of the SSR markers used in this work showed *H*_e_ values higher than 0.5, apart from Tery05, − 09 and − 13 (with values of 0.39, 0.20 and 0.22, respectively). With the only exception of Tery04 and Tery15, for most of the analysed SSRs *H*_e_ was higher than *H*_o_. It can be also observed that the whole population displayed a deficit of average *H*_o_ (0.31) compared with the *H*_e_ value (0.51) under Hardy–Weinberg equilibrium. This observation agrees with the positive value of the Wright’s fixation index (*F*_w_) estimated for all analysed SSR markers over the whole population (*F*_w_ = 0.41). The SSR markers Tery12 and Tery13 showed *F*_w_ values close to 1.0 (0.81 and 0.85, respectively), suggesting that their alleles were considerably fixed in the population.

**Table 2:** Collection data of *T. erytreae* populations.

Country	Province	Location	Date of collection	Geographical coordinates	Elevation (m a.s.l.)	Host plant
SPAIN	Gran Canaria	Valleseco	23/04/2019	28°03′44.1″N, 15°34′29.9″W	801	Citrus spp.
		Los Rodeos	03/10/2018	28°28′33.9″N, 16°20′31.4″W	660	Citrus spp.
	Tenerife	Orotava	26/09/2018	28°23′55.6″N, 16°30′19.9″W	354	Citrus spp.
		Tacoronte	26/09/2018	28°29′34.5″N, 16°24′57.4″W	370	Citrus spp.
	Pontevedra (Galicia)	Aldán	27/09/2018	42°16′52.7″N 8°49′14.2″W	9	*Citrus* spp
		Areeiro	27/09/2018	42°33′00″N 8°45′24″W	62	*Citrus* spp
		Pontevedra	24/09/2018	42°25′57.6″N 8°38′50.8″W	18	*Citrus* spp
		Portonovo	18/08/2015	42°23′24.4″N 8°49′44.9″W	22	*Citrus* spp
Portugal	Madeira	Camacha (Santa Cruz)	26/05/2019	32°40′22.1″N 16°50′49.8″W	648	*Citrus lemon*
		Quebradas (São Martinho)	22/10/2019	32°38′53.3″N 16°57′42.2″W	113	*Casimiroa edulis*
		Moreno (Ribeira Brava)	10/12/2019	32°40′37.4″N 17°03′22.3″W	214	*Citrus lemon*
		Poiso (São Vicente)	09/05/2019	32°48′01.3″N 17°02′38.9″W	85	*Citrus sinensis*
	Porto Santo	Farrobo	03/04/2019	33°04′40.7″N 16°20′54.0″W	90	*Citrus sinensis*
	Porto	Vairão (Vila do Conde)	12/12/2019	41°19′43.3″N 8°40′34.9″W	86	*Citrus lemon*
	Aveiro	São Vicente de Pereira Jusã (Ovar)	26/05/2019	40°52′57.1″N 8°31′46.2″W	151	*Citrus lemon*
	Coímbra	Paião (Figueira da Foz)	22/10/2019	40°04′08.5″N 8°48′25.8″W	88	*Citrus lemon*
	Lisbon	Areeiro (Lisbon)	10/12/2019	38°44′41.1″N 9°08′12.9″W	69	*Citrus lemon*
		Barreiralva (Mafra)	09/05/2019	38°58′38.0″N 9°20′03.7″W	209	*Citrus lemon*
		Ribamar (Lourihnã)	03/04/2019	39°00′39.6″N 9°24′39.3″W	36	*Citrus lemon*
	Setúbal	Sobreda (Almada)	12/12/2019	38°38′59.6″N 9°11′25.3″W	56	*Citrus lemon*
Kenya	Homa Bay	Homa Bay	27/11/2019	0°41′21.0″N 34°18′43.0″E	1.245	*Citrus lemon*
South Africa	Mpumalanga	Nelspruit	27/09/2017	25°22′35.7″S 30°32′33.0″E	971	*Citrus* spp
	Gauteng	Pretoria	06/10/2017	25°45′7.79″S 28°13′28.20″E	1.350	*Citrus lemon*
	Limpopo	Tzaneen	05/10/2017	23°50′13.7″S 30°09′37.8″E	733	*Citrus lemon*

**Table 3:** Statistical summary of the diversity of *T. erytreae* SSR markers.

SSR locus	*N*_*a*_	*H*_*e*_	*H*_*o*_	*F*_w_
Tery04	6	0.60	0.57	0.05
Tery05	6	0.39	0.20	0.48
Tery06	4	0.50	0.28	0.45
Tery08	12	0.72	0.34	0.54
Tery09	3	0.20	0.15	0.24
Tery10	7	0.60	0.29	0.52
Tery11	20	0.77	0.28	0.64
Tery12	7	0.54	0.10	0.81
Tery13	2	0.22	0.03	0.85
Tery15	3	0.58	0.84	− 0.44
Multi-locus	7	0.51	0.31	0.41

Mean values are represented in the table. *N*_a_, allele number; *H*_e_ and *H*_o_, expected and observed heterozygosity, respectively; *F*_w_, Wright's fixation index over the whole population.

### Population structure based on *T. erytreae* SSR data

**Figure 2:**
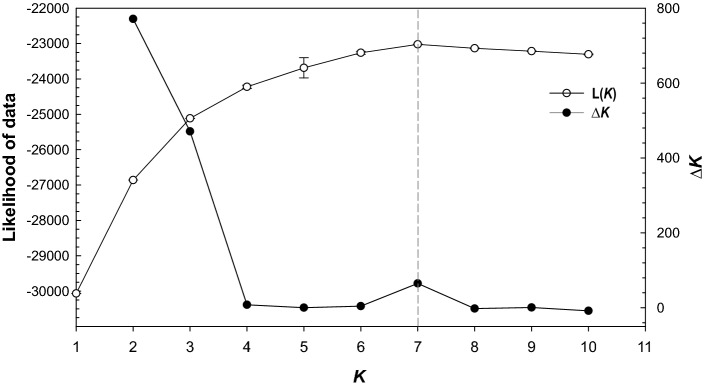
Inference of the number of unique genetic clusters (*K*) from structure simulations derived from ten SSR markers. Diagrams of posterior probability of SSR data were obtained according to the methods of Evanno *et al*^36^ and Pritchard *et al*^37^. The likelihood of data given *K* (ln Pr(X|*K*), in open circles) and Δ*K* (the standardised second order rate of change of the likelihood function with respect to *K*, in bold circles) are plotted as functions of *K*. Error bars of the ln Pr(X|*K*) indicate standard deviations, but they are too small to be seen in the plot.

**Figure 3:**
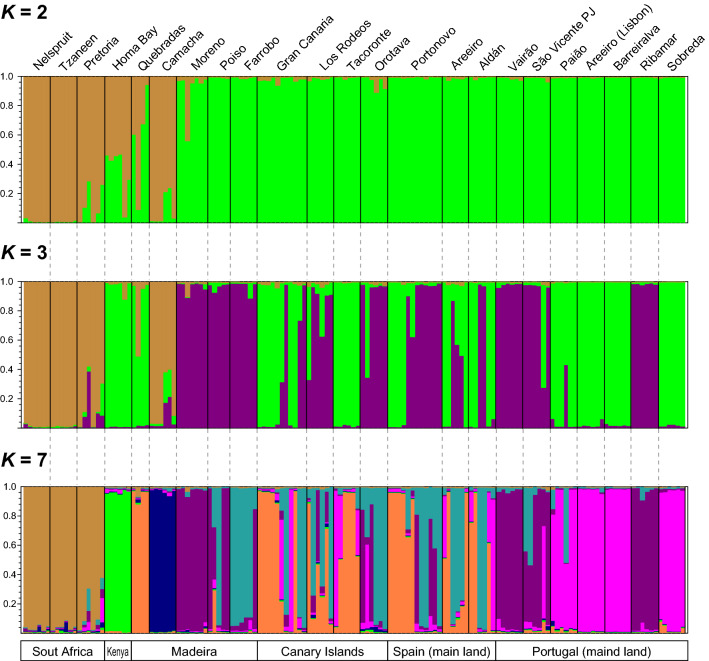
Bayesian clustering analysis of individuals genotyped with ten SSR markers in 23 populations of *T. erytreae* sampled in Africa, Spain, and Portugal. The assignment of individuals to genetic clusters inferred from STRUCTURE^37^ simulations are based on average membership coefficient (*Q*). Estimated membership fractions for each individual and population are shown for *K* = 2, 3 and 7. Selection of the number of clusters was based both on the *K* value at which the likelihood distribution began to decrease and the peak values of Δ*K*. Each individual is represented by a single vertical bar, with the colouring of each bar represents the stacked proportion of assignment probabilities to each genetic cluster. For *K* = 7, clusters 1, 2, 3, 4, 5, 6 and 7 are shown in green, beige, purple, pink, light blue, orange, and dark blue, respectively. Black vertical lines separate sample sites. Labels identify *T. erytreae* populations from old invaded areas in Africa, and newly invaded areas in the Iberian Peninsula and the Macaronesia.

To assess the differentiation and genetic diversity among the local populations of *T. erytreae* sampled in newly invaded areas from Spain and Portugal, including Madeira and the Canary Islands, and those from the previous invaded areas in Africa (South Africa and Kenya), we used a Bayesian clustering method to analyse the SSR multi-locus genotyping data. The STRUCTURE analysis according to the method of Δ*K*^36^ showed that the overall genetic profile of all the individuals sampled could be described with two or three different hypothetically original populations corresponding to the highest Δ*K* values (Fig. 2). It means that the most likely values of genetic clusters (*K*) are 2 or 3. Nevertheless, Pritchard’s method^37^ showed a posterior probability of data at *K* = 7 (Fig. 2). The estimated likelihood distribution increased from *K* = 1 to *K* = 7, and then started to decrease. This implied that seven was the smallest value of *K*, which was the most likely number of inferred populations in our data set. Interestingly, the value of *K* at which the likelihood distribution reached its maximum coincided with a further peak value of the Δ*K* statistic at *K* = 7, suggesting a more complex hierarchical structure of the *T. erytreae* populations (Fig. 2). In consequence, we plotted the clustering results for *K* = 2, *K* = 3 and *K* = 7 (Fig. 3). Furthermore, we considered an initial structure of two populations (*K* = *2*) as was suggested by the method of Δ*K*^36^ whereby most of the analysed individuals were classified with high probability (*Q* > 0.90) in two clusters (Fig. 3). Cluster 1 (in green) was exclusively formed by individuals from newly invaded areas in Spain and Portugal, including those from the archipelagos of Madeira and the Canary Islands. On the other hand, Cluster 2 (in beige) was mainly comprised of individuals from Africa, but also included individuals from Camacha (Madeira). The exception to this pattern involved three locations in Madeira (Quebradas, Camacha and Moreno), Pretoria (South Africa), and Homa Bay (Kenya), where almost all individuals consistently had significant membership in both clusters. Looking at *K* = 3 plot, the Bayesian clustering analysis resolved Cluster 1 into two by reassigning some individuals to Cluster 3 (in purple). Almost of all individuals from Moreno, Poiso, and Farrobo (in Madeira and Porto Santo, respectively) were entirely reassigned to Cluster 3 along with several individuals from the Canary Islands and Galicia (Spain). In addition, individuals from Vairão (Porto) and São Vicente de Pereira Jusã (Aveiro) (both in the northwest coast of Portugal) were also assigned to Cluster 3, while those individuals sampled from southern locations up to Sobreda (Setúbal) were assigned to Cluster 1. The exceptions to this pattern were the individuals from Ribamar (Ericeira), which were assigned to Cluster 3. Most notably, samples from Kenya were genetically different from those of South Africa and grouped in Cluster 1. At *K* = 7 the population structure scenario was more hierarchical, but 73% of all individuals (108 out from 147) could be assigned to one of the seven clusters with more than 90% probability (*Q* > 0.9). The assignment of half of the remaining individuals (21 out of 39) could be done with more than 70% probability (*Q* > 0.7). Among the different groups, Cluster 1 (in green) and 2 (in beige) are restricted to the populations of South Africa and Kenya, respectively, with almost no presence of individuals from any of the newly invaded areas. Clusters 3 (in purple) and 4 (in pink) are mostly exclusive to the individuals from Madeira and Portugal mainland, although with some membership in the Canary Islands and Galicia. Cluster 5 (in light blue) and Cluster 6 (in orange) are represented by individuals from Madeira, the Canary Islands and Galicia, while the individuals from Camacha (Madeira) –the only ones that were collected from *Casimiroa edulis* La Llave & Lex. (Rutacea: Toddalioideae)—form exclusively Cluster 7 (in dark blue). Remarkably, *Q* fractions corresponding to Cluster 7 are present in the individuals from Nelspruit, Tzaneen, and some in Pretoria.

### Genetic diversity analysis using *T. erytreae* SSR allelic data

**Figure 4:**
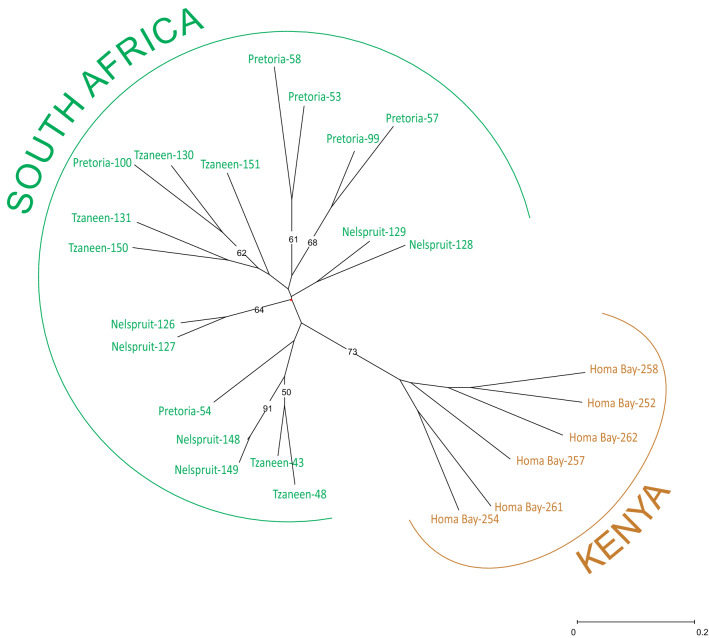
NJ consensus tree showing the phylogenetic relation between analysed individuals from *Trioza erytreae* populations sampled in South Africa and Kenya. Consensus tree is the result of 10,000 iterations of genetic allelic data obtained for the ten SSR markers selected in this work. Bootstrap values over 50% are indicated.

**Figure 5:**
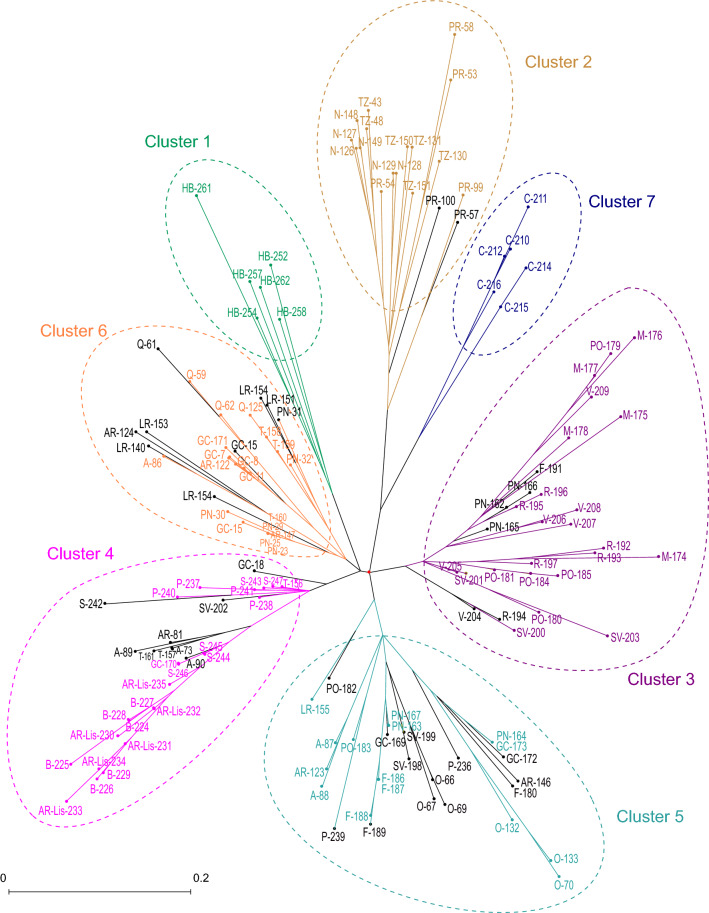
NJ tree under topological constraints showing the phylogenetic relation among the populations of *T. erytreae* sampled in newly invaded areas in Spain and Portugal and those from old invaded areas in Kenya and South Africa, respectively. Dendogram is the result of 10,000 iterations of allelic data obtained for the ten SSR loci developed in this work. Structure of the tree inferred from allelic data of individuals with *Q* > 0.7 according to STRUCTURE^37^ was used as initial tree, and the remaining individuals were positioned (constraint) on this previous topology. **Spain:** Aldán (A), Areeiro (AR), Gran Canaria (GC), Los Rodeos (LR), Oratava (O), Portonovo (PN), Tacoronte (T). **Portugal:** Areeiro-Lisbon (AR-Lis), Barreiralva (B), Camacha (C), Farrobo (F), Moreno (M), Paião (P), Poiso (PO), Quebradas (Q), Ribamar (R), Sobreda (S), São Vicente de Pereira Jusã (SV), Vairão (V). **South Africa:** Nelspruit (N), Pretoria (PR), Tzaneen (TZ). **Kenya:** Homa Bay (HB). Genetic clusters for *K* = 7 are indicated. Admixed individuals with *Q* < 0.7 are shown in black.

The genetic diversity of *T. erytreae* populations was also assessed by means of a distance-based clustering method. The scored SSR allelic data obtained from the ten SSR loci developed in this study were used to calculate a genetic dissimilarity matrix and to compute a Neighbor Joining (NJ) tree. A preliminary dendogram constructed using only the African populations of *T. erytreae* showed that the individuals from South Africa grouped together into a single cluster clearly separated from the Kenyan population. The robustness of the tree clustering was supported by the high bootstrap values obtained for nearly all branches (Fig. 4). To confirm the results obtained from the structure analysis a NJ tree under topological constraints was inferred using as initial tree the population structure of individuals from all the sampled areas with *Q* > 0.7. The remaining individuals were positioned (constraint) on that previous topology. Inspection of the constrained tree topology revealed seven clusters that were in congruence with the structural population at *K* = 7 suggested by the STRUCTURE analysis (Fig. 5). It is noteworthy that Cluster 7 emerged as a paraphyletic group in the base of African Cluster 2. The cluster assignments of individuals with low membership coefficients (*Q* < 0.7) performed well in our distance-based clustering analysis.

### Phylogenetic analysis using mtDNA-based barcoding

The maternal phylogenetic relationship among the *T. erytreae* individuals collected in this study was assessed using mitochondrial DNA (mtDNA) barcoding, the sequence analysis of nucleotide variations in the 5’ region of the *Cytochrome C Oxidase I* gene (*COI*)^38^. Comparison of the nucleotide sequence of COI barcode fragments from this study with other *T. erytreae* GenBank accessions demonstrated that all sequences are highly conserved (Supplementary Data 3). With the only exception of the fragments amplified from Kenyan individuals, all remaining sequences showed absolute identity (100%) to the GenBank accession numbers that we previously deposited in the GenBank^39^, corresponding to COI barcode sequences from *T. erytreae* individuals collected in the Canary Islands (MK285551-MK285553), Galicia (MK285548-MK285550), Madeira (MK285558) and South Africa (MK285554-MK285557, MK285559, MK285560) (Supplementary Data 4). Alignment of sequences amplified in this study from individuals collected in Homa Bay (Kenya) shared absolute identity with those extracted from the entire mitochondrion genome sequences from Eritrea and Uganda^27^, and 97–98% identity with those COI barcode fragment sequences from individuals from other locations in Kenya^39^.

**Figure 6:**
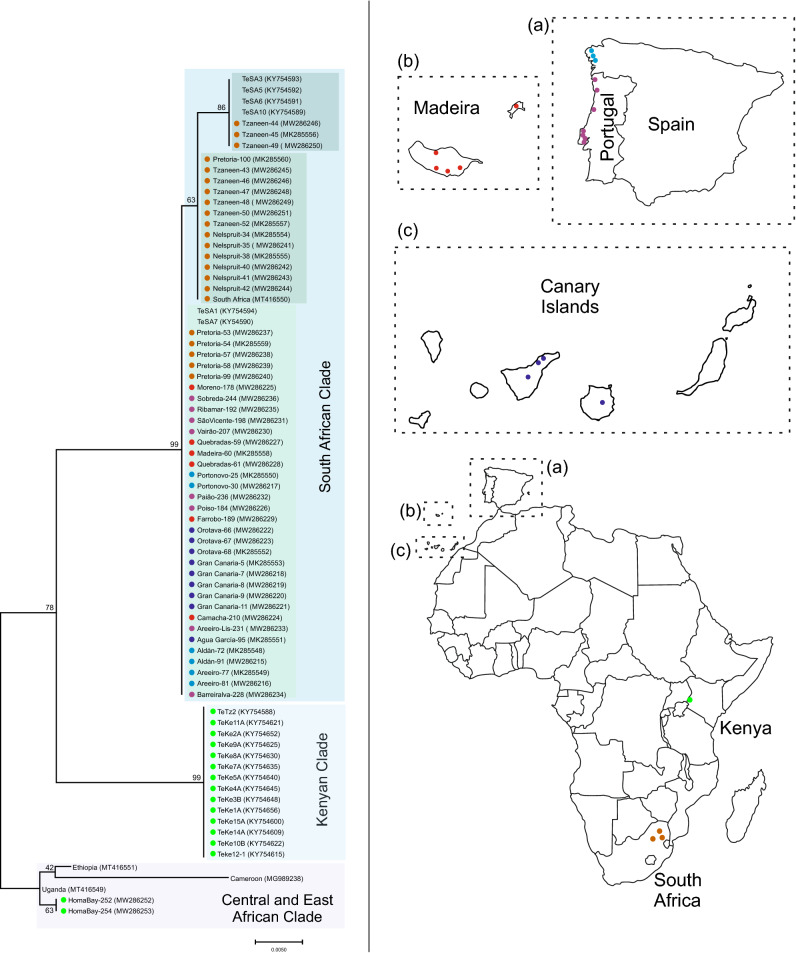
Phylogenetic tree based on COI barcode sequences of *Trioza erytreae* individuals from invaded areas in Spain and Portugal, and African local populations. The evolutionary history was inferred by means of the Maximum Likelihood method and Tamura-Nei model^41^. The analysis involved 76 nucleotide sequences, including those generated from this study and 37 available GenBank accessions of *T. erytreae*. The tree with the highest log likelihood (− 1037.95) is shown. The percentage of trees in which the associated taxa clustered together is shown next to the branches. The tree is drawn to scale, with branch lengths measured in the number of substitutions per site. Codon positions included were 1st + 2nd + 3rd + noncoding. There was a total of 657 positions in the final dataset. Evolutionary analyses were conducted in MEGA X^42^. GenBank accession numbers are shown in brackets. Colour dots beside the COI accessions generated in this study correspond to the sampling location in the map: Kenya (green), South Africa (brown), Madeira (red), Canary Islands (deep purple), Portugal (magenta) and Spain (blue) main land. Maps were taken and manipulated from www.outline-worldmap.com.

The nucleotide sequences of the COI barcode fragments generated in this study (n = 39) and some previously deposited in the GenBank (n = 37) were used to analyse the maternal phylogenetic relationship of *T. erytreae* populations that have invaded Spain and Portugal, with those from South Africa and Kenya (Fig. 6). From all COI sequences used in this study, 38.2% were obtained from Spain and Portugal (including Madeira and the Canary Islands), 36.8% from South Africa, 19.7% from Kenya, and 5.3% from other African countries (Cameroon, Ethiopia, Tanzania and Uganda). In accordance with the high level of identity of their COI barcode nucleotide sequences, the NJ tree generated from these sequences showed that the individuals from Spain and Portugal, including those from Madeira and the Canary Islands, formed a monophyletic group with the individuals from South Africa (Pretoria, Nelspruit, and Tzaneen). Our phylogenetic analysis reveals a clear differentiation between this monophyletic group and the individuals from Homa Bay (Kenya), as well as from those individuals previously reported in other locations in Kenya^39^. It was observed that the individuals from Spain and Portugal formed a paraphyletic group with those from Pretoria (Fig. 6), as the remaining South African individuals from Nelspruit and Tzaneen formed a separated clade. Furthermore, our analysis demonstrated the presence of two different *T. erytreae* lineages in Tzaneen, as most of their individuals formed a paraphyletic group with those from Nelspruit, while the remaining formed a clade with four individuals from West Acres (South Africa)^39^. The few exceptions to this observation were three South African individuals, one from Pretoria (Pretoria-100), and two from West Acres (TeSA1 and TeSA7), which may correspond to migrants from Nelspruit or Tzaneen, and Pretoria, respectively. In a sister clade position to the South African clade, the GenBank accessions of Kenyan and Tanzania COI sequences^39^ included in this phylogenetic analysis formed a monophyletic group. The COI barcode sequences from Homa Bay (Kenya) clustered separately as an outgroup in a different clade with the corresponding fragment extracted from the mitochondrion genome sequences from Ethiopia and Uganda (MT416551 and MT416549, respectively)^27^, and Cameroon (MG989238)^40^ present in GenBank.

## Discussion

It is well known that SSR markers are useful to study population genetics and phylogeography in insect species and have been proven to be an efficient molecular tool to assist in the optimisation of integrated pest management programs^43,44^. However, although the contribution of SSRs in studies of population diversity and structure is undeniable, their comprehensive characterisation and development as molecular markers are only possible after genome sequences are available. In Hemiptera, microsatellites have been characterized for several invasive and devastating pests, including the Asian citrus psyllid *D. citri*^45^. The recent sequencing of *T. erytreae* genome has made this first report of a comprehensive identification and distribution analysis of SSRs for this invasive and devastating citrus pest possible. We found that the total number and frequency of *T. erytreae* SSR markers (428,342 SSRs and 1 SSR/1.09 Kb) are about 3.6 and 3.8 times lower, respectively, compared to *D. citri* (1,547,487 SSRs and 3.8 SSR/1 Kb)^38^. The estimated frequency in other Hemiptera range from 0.16 for a vector of Chagas disease *Rhodnius prolixus* Stål (Hemiptera: Reduviidae), to 33 SSRs/Kb for the rice brown plant hopper *Nilaparvata lugens* Stål (Hemiptera: Delphacidae)^45^. Since the genome of *T. erytreae* is not yet fully annotated, we could not estimate the total number of SSRs in exons, introns or intergenic regions, but it was possible to get information for most of the SSR loci selected for further analysis. When comparing the number of various classes of SSRs in *T. erytreae*, we found that our data were in accordance with the averages estimated for other insect genomes^38^. The average of di- and trinucleotide SSRs is significantly higher than those observed for longer repeat types. Among the grouped motif pairs in *T. erytreae*, we found that CT/AG and GA/TC were predominant, while GC/GC and CG/CG were the least frequent di-nucleotide motifs. These data agree with published results in other insect species, including members of the order Hemiptera^45^.

In our study we developed ten informative microsatellites markers to determine the genetic variability and to estimate the gene flow and the structure of *T. erytreae* populations that have invaded Spain and Portugal. The Wright’s fixation index (*F*_w_) for all loci was higher than zero (0.41) considering the whole individuals sampled, indicating an overall heterozygote deficiency. This also suggested a slight degree of inbreeding among individuals within local populations. Our results show that the *T. erytreae* individuals analysed in this study are structured in seven genetic clusters (*K* = 7).

Although *T. erytreae* was recorded in the Iberian Peninsula in 2014, just 7 years ago^17^, our results suggest that genetic structure was already present among the newly invaded areas in the west coast of the Iberian Peninsula, from Galicia (Spain) to Setúbal (Portugal), between 2015 and 2019. Since *T. erytreae* was spotted in Galicia 12 years after of being reported in the Canary Islands, and that individuals from both locations share a very similar genetic structure, the Canary Islands emerge as the most likely source of the *T. erytreae* that invaded the north-western coast of the Spainish mainland. The little genetic homogeneity found in local populations suggests the presence of different colonizing lineages. In this regard, there is strong and growing evidence suggesting that multiple introductions, complex global movement, and population admixture contribute to increase the genetic diversity in invasive insect species, and that mixing of different lineages may contribute to rapid evolution and to invasiveness^46^.

Since the outbreak of *T. erytreae* was first reported in Madeira in 1994^13^ and 8 years later (2002) in the Canary Islands^14^, it is possible that colonization of the latter archipelago has derived, at least in part, from Madeira. According to the genetic clusters identified in both archipelagos, the colonisation of the Canary Islands could have originated mainly from Quebradas and Farrobo (in the islands of Madeira and Porto Santo, respectively), and in a minor extension from Moreno and/or Poiso (both in Madeira). Our study shows that *T. erytreae* individuals from Madeira, the Canary Islands and Galicia are genetically more diverse compared to those from the sample locations in the coast of Portugal, South Africa and Kenya. Conversely to the high genetic diversity observed in Galicia, the local populations of *T. erytreae* sampled along the coast of Portugal seem relatively homogeneous with only two main genetic clusters. It is probable that the expansion of *T. erytreae* observed in recent years along the coast of Portugal^18^ has had its origin in the invaded locations of Madeira, but we cannot rule out that it may have also been derived from Galicia. Remarkably, our distance-based clustering analysis of SSR allelic data suggest that the Camacha linage may have been derived from South Africa, somewhere in or around the triangle made up for the localities of Pretoria, Nelspruit, or Tzaneen, as the emerging position of the Cluster 7 in the base of the Cluster 2 supports this hypothesis.

The genetic diversity of *T. erytreae* populations from South Africa and Kenya seems different from each other. Moreover, their genetic diversity is also different from those populations of *T. erytreae* sampled in Spain and Portugal. Therefore, we cannot conclude from our study that the *T. erytreae* populations that invaded the Macaronesia and the Iberian Peninsula derived directly from one of these two African countries. Recently, a study on the intraspecific diversity of *T. erytreae* assessed by COI barcoding suggested that the populations of this species that have been found in Europe is most likely originated from South Africa^27^. The study that was strongly biased towards COI barcode accessions from Kenya with 74.2% of all sequences, compared to 14.6% from South Africa and only 7.9% from Spain and Portugal, and could not exclud the possibility of a Kenyan origin. However, according to our STRUCTURE analysis, the presence in Pretoria (South Africa) of discrete membership fractions of the genetic clusters detected in the new invaded areas in Spain and Portugal suggests that the *T. erytreae* individuals that have invaded Europe may have their origin in South Africa. Our phylogenetic analysis using mtDNA barcoding also supports this hypothesis, as the individuals from Spain and Portugal (including those from the Canary Islands and Madeira) formed a monophyletic group with those individuals from Pretoria, Tzaneen and Nelspruit, suggesting that the *T. erytreae* individuals that invaded the Iberian Peninsula have their maternal origin somewhere in the northeast of South Africa. Furthermore, the fact that *T. erytreae* populations in the Canary Islands have been drastically reduced by its natural enemy *Tamarixia dryi* (Del Guercio) (Hymenoptera: Eulophidae), a highly specific parasitoid of *T. erytreae* imported from Pretoria^21,22^ and released in the archipelago in 2018^23^, also supports the idea that the *T. erytreae* individuals that invaded the Macaronesia and the Iberian Peninsula have derived from the northeast of South Africa near Pretoria. Nevertheless, considering that Pretoria (Gauteng) is not a citrus growing area, it could be possible that the individuals of *T. erytreae* sampled in Pretoria could have derived from the neighbouring province of Limpopo, the largest citrus producing area in South Africa^47^.

A similar spatial pattern of invasion was observed in the case of the black citrus aphid *Aphis citricidus* (Kirkaldy) (Hemiptera: Aphididae), the main vector of citrus tristeza virus (CTV). This aphid species first invaded Madeira in 1994^48^, and 8 years later the north-western region of the Iberian Peninsula^49^. This similar invasive behaviour suggests a common pathway for both citrus pests. *T. erytreae* and *A. citricidus* are oligophagous species, with *Citrus* species as principal host plants but also feeding on other Rutaceae, and that the importation of citrus plants from non-European countries is forbidden in the EU. Taking all of this into consideration, we hypothesise that the introduction pathway of *T. erytreae* in the Macaronesia and Iberian Peninsula could be related to the commercial trade or transportation by travellers of ornamental Rutaceae. In the case of *T. erytreae*, another possibility was recently suggested which showed that the adults of the psyllid can survive several days on citrus fruits in low temperatures^20^.

## Materials and methods

### *Trioza erytreae* samples collection and total DNA extraction

A total of 147 T*. erytreae* individuals from 23 locations across Spain, Portugal, Kenya, and South Africa were collected between 2017 and 2019 (Table 2). Geographical coordinates and host plants species were recorded. All samples were collected from citrus genus except one sample that was obtained from white sapote *Casimiroa edulis* La Llave & Lex. in Madeira, Portugal. Upon sampling, individuals were stored in 70% ethanol at 4˚C for preserving DNA integrity. Total DNA (mix of genomic and mitochondrial DNA) was isolated from individual insects following a modified Salting Out method^50^.

### DNA barcoding and phylogenetic analysis of COI

Total DNA (10 ng) of selected *T. erytreae* individuals was used as template for PCR amplification of a 714 bp fragment of the 5’-coding region of the mitochondrial *Cytochrome C Oxidase I* gene (*COI*; Gene ID: 37,507,472), from positions + 6 to + 719 with respect to the start codon. The COI barcode fragment was amplified using the specific primers Te-6U30 and Te-720L26, previously published by us^22^. PCR fragments were bi-directional sequenced using the same both primers. COI barcode fragments were sequenced by capillary electrophoresis using a 3130XL Genetic Analyzer (Applied Biosystems, Carlsbad—California, USA), at the DNA Sequencing Unit of the IBMCP (Valencia, Spain). Sequences were analysed and trimmed to remove primer sequences, using the Sequencer DNA Sequence Analysis Software v4.9 (Gene Codes Corporation, Michigan, USA). Forward and reverse high-quality reads obtained for each *T. erytreae* individual were assembled into consensus sequences and submitted to the GenBank public sequence repository. Multiple alignment of nucleotide sequences of COI barcode fragments was performed in MEGAX^42^ using the built-in ClustalW alignment interface. For each COI barcode sequence, a fragment of 657 bp (from positions + 36 to + 692 with respect to the ATG) was used for multiple sequence alignment. The phylogenetic analysis was also carried out on MEGA X using the Maximum Likelihood Method based on the Tamura-Nei model^41^. The reliability of the constructed tree was evaluated using a bootstrap test of 10,000 replicates. Initial tree for the heuristic search were obtained automatically by applying the Maximum Parsimony method. A discrete Gamma distribution was used to model evolutionary rate differences among sites (5 categories [+ G, parameter = 0.0500]).

### Genome mining for SSRs discovery

A genome sequence draft of *T. erytreae*, obtained in collaboration with the Genome Assembly and Annotation Team (CNAG-CRG, Barcelona—Spain), with an estimated size of 763 Mb (Rojas-Panadero et al.,* unpublished data*), was mined for SSRs by means of the GMATA^35^ software (Genome-wide Microsatellite Analysing Toward Application, http://sourceforge.net/p/GMATA). The parameters of motif unit length were set to a minimum of two (excluding mononucleotide motifs) and maximum of ten nucleotides repeats, while the minimum of in tandem repeat copies of detected motifs (from di- to octa-nucleotides) was set to three.

**4.4 PCR amplification and Genotyping of SSR loci**. Excluding mononucleotides motives, SSRs of three or more in tandem repeated motifs were targeted to design primers for PCR amplification. Primer pairs with sufficient flaking sequence to amplify genomic SSR loci between 250 and 600 bp were designed in Primer Analysis Software Oligo 7.6 (MedProbe, Colorado, USA), using very high constrain parameters with Tm ranged between 58 and 60 °C. Amplicons sequence were blasted via BlastN against the *T. erytreae* draft genome sequence using the CLC Genomics Workbench v.9.5.3 (CLCbio, Aarhus, Denmark), to determine whether PCR primers would result in the amplification of single DNA fragments for each SSR loci. Finally, primer pairs were tested for amplification in PCR reactions using the Phire Hot Start II DNA Polymerase (Thermofisher Scientific; Vilnuis, Lithuania). PCR reactions were carried out in a total reaction volume of 20 µl containing 1X Phire Reaction Buffer (provided with 1.5 mM MgCl_2_), 200 μM of all four dNTPs, 500 μM of forward and reverse primers, 0.4U of DNA Polymerase, and 20 ng of total DNA. Cycled conditions were set as: initial denaturation at 95 °C for 30 s, followed by 35 cycles of denaturation at 94 °C for 5 s, annealing at 60 °C for 5 s and extension at 72 °C for 10 s; and 10 min of extension at 72 °C. The PCR products were stained with gelRed and then resolved through a 2% agarose gel and visualised under UV light. Genotyping of selected SSR loci was performed on a capillary genetic fragment analyser. PCR reactions were set up as mentioned above with the only exception that forward primers labelled with wellRED fluorescent dye were used instead of unlabelled oligonucleotides. Denaturation and capillary electrophoresis were carried out on a CEQ™ 8000 Genetic Analysis System (Beckman Coulter Inc; Fullerton, USA), using linear polyacrylamide gel according to the manufacturer’s instructions. The PCR amplified fragments were sized based on 400 or 600 bp DNA size standards (Beckman Coulter Inc; Fullerton, USA). Genetic analysis system software GenomeLab™ GeXP v10.0 was used for data collection and analysis, and subsequently to estimate the genetic diversity.

### Genetic diversity analysis of *T. erytreae* populations

Population diversity organization was analysed with the bioinformatic software DARwin v6^51^ (Dissimilarity Analysis and Representation for Windows, http://darwin.cirad.fr/darwin). For each SSR locus, amplicons were scored as allelic data to calculate the genetic dissimilarity matrix using the simple matching dissimilarity index between pairs of accessions (units). Neighbour-Joining (NJ) tree was computed using the obtained dissimilarity matrix. The robustness of the consensus tree branches was tested using 10,000 bootstrap iterations.

### Population genetic parameters and genetic structure analysis based on the SSR markers data

Two analyses were conducted to examine the populations of *T. erytreae* that invaded the Macaronesia (Madeira and Canary Islands) and the Iberian Peninsula (Table 1). Polymorphism analysis of the selected SSR loci was carried on the sampled *T. erytreae* local populations with factorial discriminant analysis performed in GENETIX^52^ (https://kimura.univ-montp2.fr/genetix/), a multivariate analysis approach that uses no priori genetic assumptions for relationships between allelic differences and genetic distances. By means of GENETIX, descriptive statistics of SSR allelic data, including the mean number of alleles per locus (*N*_a_), the expected (*H*_e_) and observed heterozygosity (*H*_o_), Wright's fixation index over the whole population (*F*_w_) and the inbreeding coefficient (*F*_is_) were estimated (Table 3). A Bayesian clustering analysis performed with the STRUCTURE v2.3.3 package software^37^ (http://cbsuapps.tc.cornell.edu/structure) was used to infer the genetic structure of the *T. erytreae* local populations. The software assumes a model in which there are *K* unknown populations, each of which is characterized by a set of allele frequencies at each analysed locus. The STRUCTURE analysis was done using Admixture model but allowing allele frequencies to vary independently across populations. The additional parameters used were different values of *F*_ST_ for different subpopulations, prior *F*_ST_ mean 0.01, standard deviation 0.05, and constant lambda valued was set to 1. The length of the initial burn-in period was set to 500,000 iterations of burning followed by 1,000,000 Monte Carlo Markov Chain (MCMC) repetitions in the data collection phase, with ten independent runs for each value of *K*, and the number of inferred clusters varying from one to ten. Individuals in the populations were probabilistically assigned to hypothetical original populations, or jointly to two or more populations if their genotypes indicate that they are admixed. The most likely number of *K* was jointly determined by the method of Δ*K* developed by Evanno *et al*^36^ and the Pritchard’s method^37^ based on the estimation of the *K* value at which the likelihood distribution began to decrease. The run at which *K* value yield the highest likelihood of the data given the parameters values was used for plotting the distribution of individuals membership coefficients (*Q*). Individuals analysed in this study were assigned with high probability (*Q* membership coefficient > 0.90), while the assignment of admixed individuals to the most likely population was set at *Q* > 0.70.

## Supplementary Information

Supplementary Information 1.

Supplementary Information 2.

Supplementary Information 3.

Supplementary Information 4.

Supplementary Information 5.

## Data Availability

Thirty-nine COI barcode DNA fragments generated from this study were deposited in the GenBank under the accession numbers MW286215—MW286253.
