# Supplementary material for: Insights into the origin of the invasive populations of Trioza erytreae in Europe using microsatellite markers and mtDNA barcoding approaches
Source: Sci Rep. 2021 Sep 20;11:18651. doi: 10.1038/s41598-021-97824-0 (PMC8452619; doi:10.1038/s41598-021-97824-0)
Supplement: Supplementary file 3 — Supplementary Information 3. [file 41598_2021_97824_MOESM3_ESM.pdf]

ClustalW aligment of 39 COI barcode DNA fragments generated from this study and 37 accessions retrieved from the GenBank. Coordinates are given with respect to the original nucleotide sequences, with the exception of the accessions MG989238, MT416549, MT416550, and MT416551 for which the coordinates are given with respect to the whole mitochondrial genome sequence of *Trioza erytreae*. For sequence alignment a 657 bp fragment, from positions +6 to +719 with respect to the start codon of the COI coding region, was used.

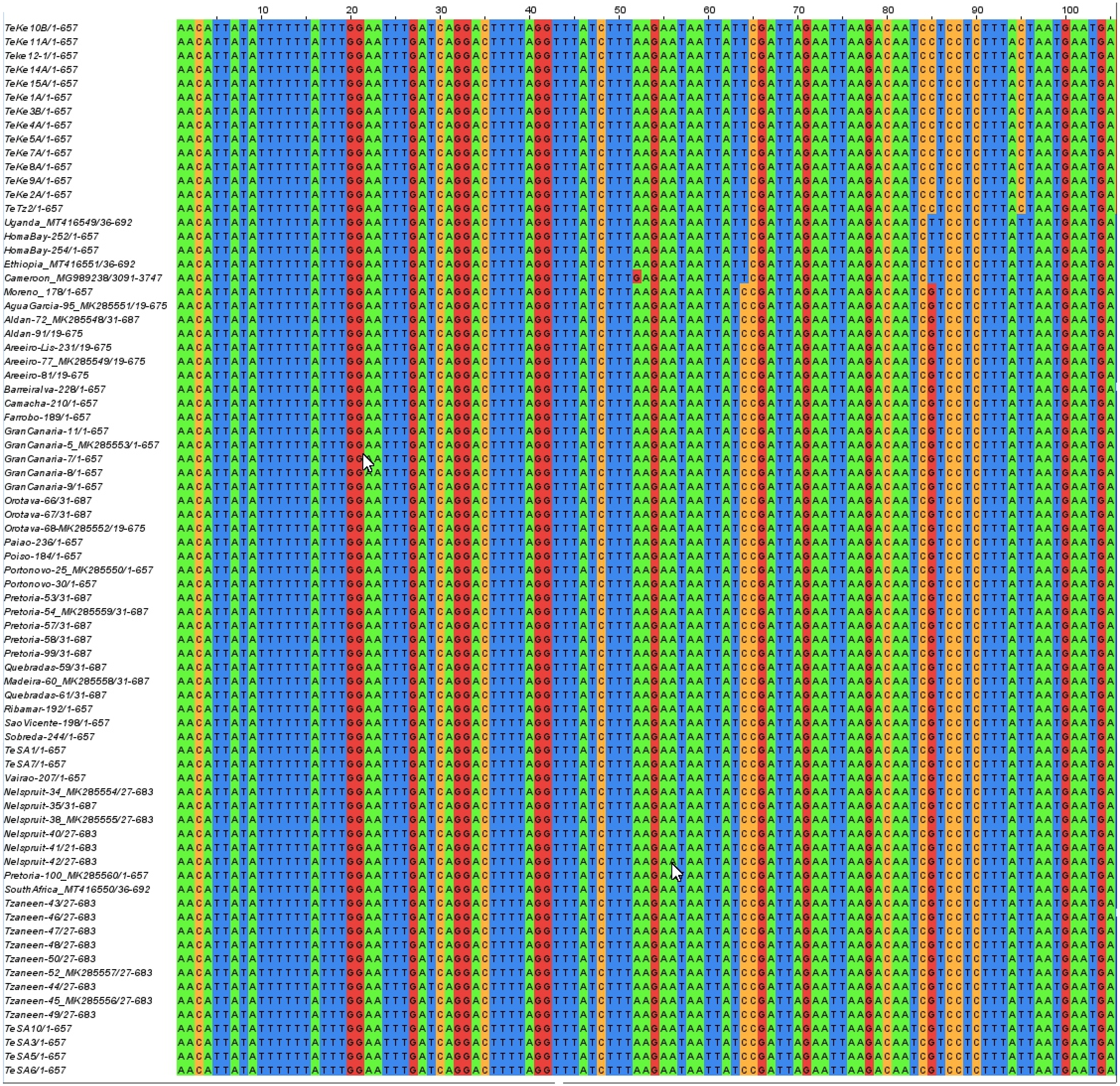

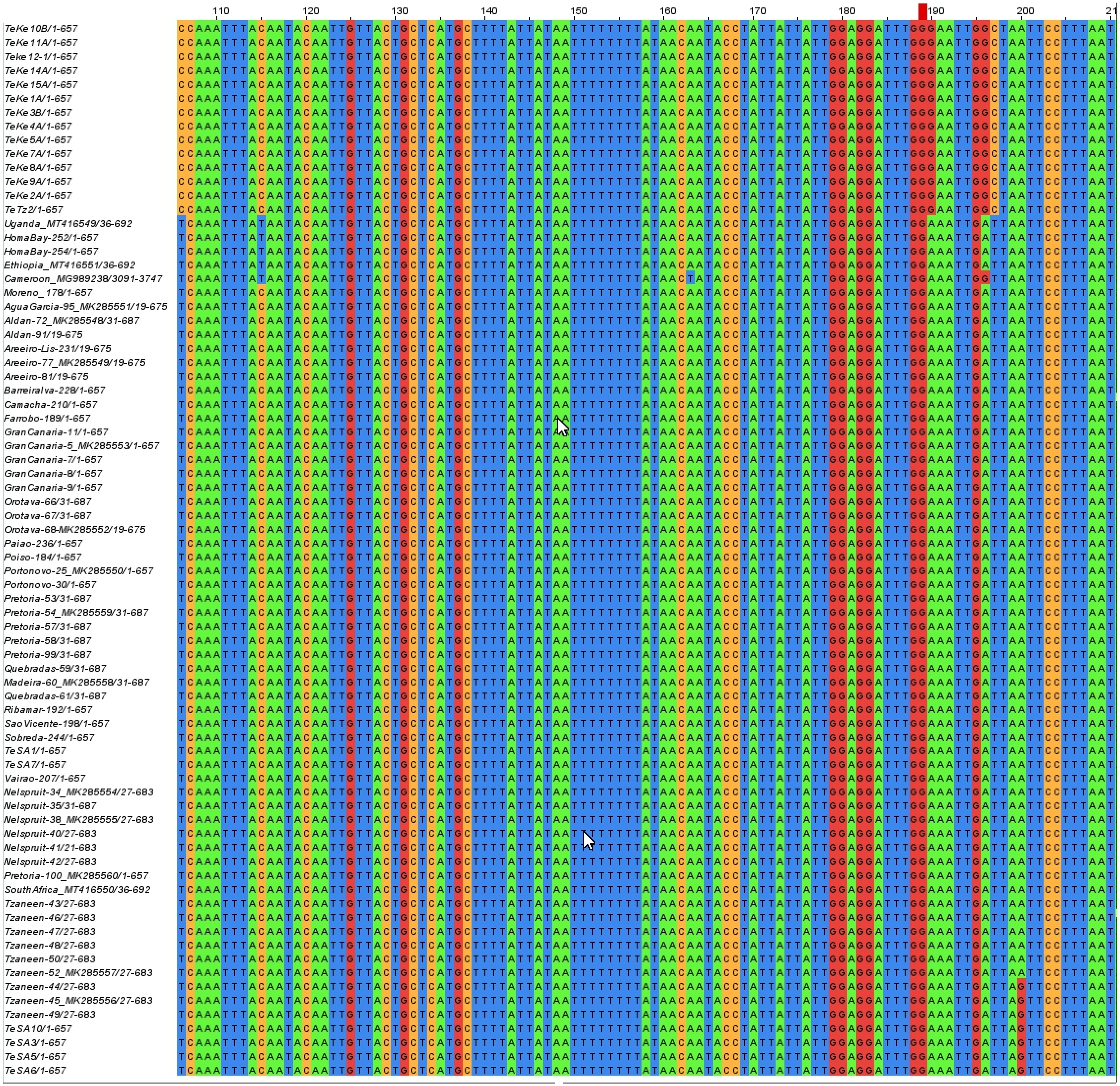
110
120
130
140
150
160
170
180
190
200
TeKe10B/1-657
TeKe11A/1-657
Teke12-1/1-657
TeKe14A/1-657
TeKe15A/1-657
TeKe1A/1-657
TeKe3B/1-657
TeKe4A/1-657
TeKe5A/1-657
TeKe7A/1-657
TeKe8A/1-657
TeKe9A/1-657
TeKe2A/1-657
TeTz2/1-657
Uganda_MT416549/36-692
HomaBay-252/1-657
HomaBay-254/1-657
Ethiopia_MT416551/36-692
Cameroon_MG989238/3091-3747
Moreno_178/1-657
AguaGarcia-95_MK285551/19-675
Aldan-72_MK285548/31-687
Aldan-91/19-675
Areeiro-Lis-231/19-675
Areeiro-77_MK285549/19-675
Areeiro-81/19-675
Barreiralva-228/1-657
Camacha-210/1-657
Farrobo-189/1-657
GranCanaria-11/1-657
GranCanaria-5_MK285553/1-657
GranCanaria-7/1-657
GranCanaria-8/1-657
GranCanaria-9/1-657
Orotava-66/31-687
Orotava-67/31-687
Orotava-68-MK285552/19-675
Paiao-236/1-657
Poiso-184/1-657
Portonovo-25_MK285550/1-657
Portonovo-30/1-657
Pretoria-53/31-687
Pretoria-54_MK285559/31-687
Pretoria-57/31-687
Pretoria-58/31-687
Pretoria-99/31-687
Quebradas-59/31-687
Madeira-60_MK285558/31-687
Quebradas-61/31-687
Ribamar-192/1-657
SaoVicente-198/1-657
Sobreda-244/1-657
TeSA1/1-657
TeSA7/1-657
Vairao-207/1-657
Nelspruit-34_MK285554/27-683
Nelspruit-35/31-687
Nelspruit-38_MK285555/27-683
Nelspruit-40/27-683
Nelspruit-41/21-683
Nelspruit-42/27-683
Pretoria-100_MK285560/1-657
SouthAfrica_MT416550/36-692
Tzaneen-43/27-683
Tzaneen-46/27-683
Tzaneen-47/27-683
Tzaneen-48/27-683
Tzaneen-50/27-683
Tzaneen-52_MK285557/27-683
Tzaneen-44/27-683
Tzaneen-45_MK285556/27-683
Tzaneen-49/27-683
TeSA10/1-657
TeSA3/1-657
TeSA5/1-657
TeSA6/1-657

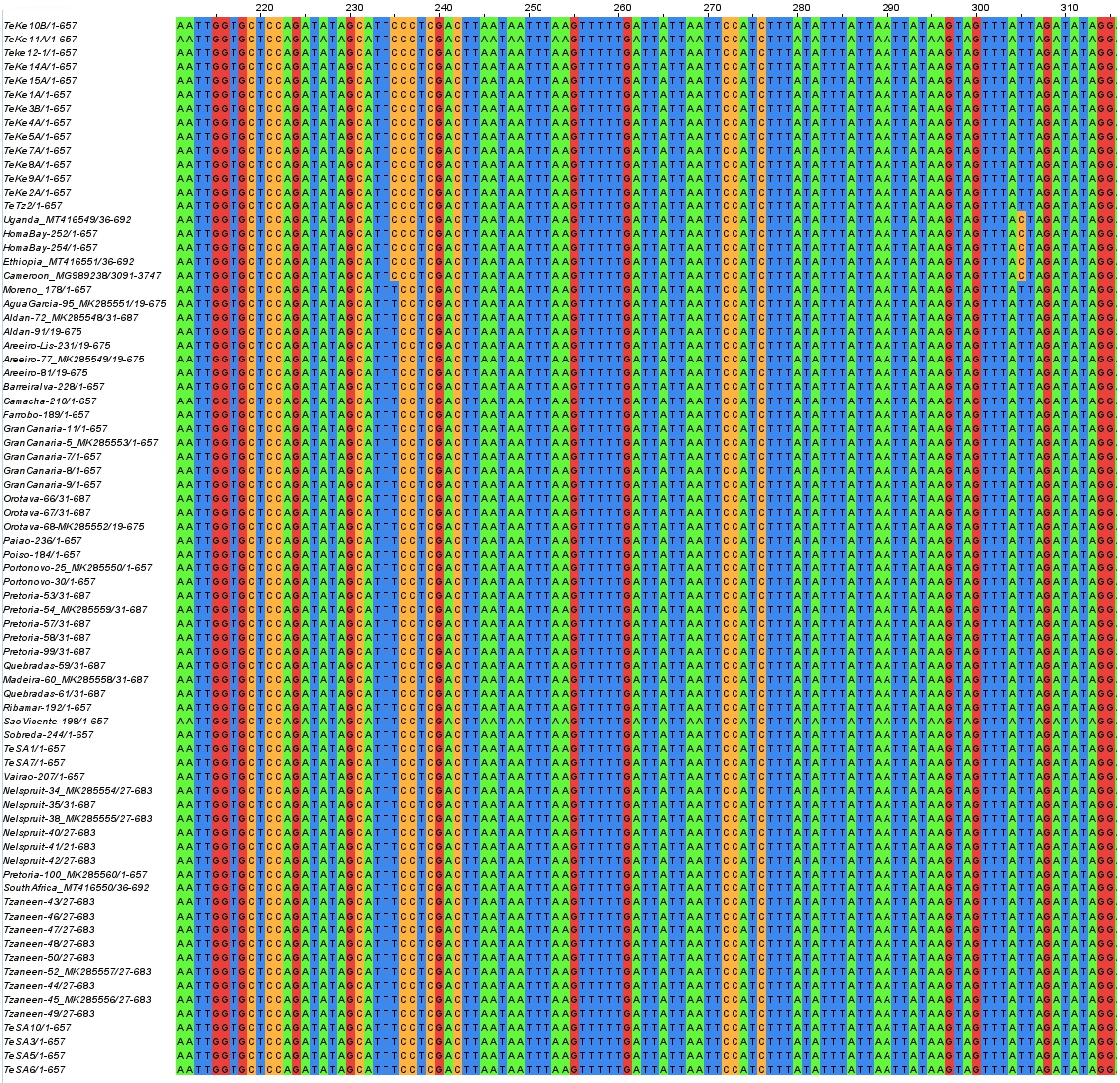
220 230 240 250 260 270 280 290 300 310
TeKe10B/1-657 AATTGGTGCTCCAGATATAGCATTCCCTCGACTTAATAATTTAAGTTTTTGATTATTAATTCCATCTTTATATTTATTAATTATAAGTAGTTTATTAGATATAGG.
TeKe11A/1-657 AATTGGTGCTCCAGATATAGCATTCCCTCGACTTAATAATTTAAGTTTTTGATTATTAATTCCATCTTTATATTTATTAATTATAAGTAGTTTATTAGATATAGG.
Teke12-1/1-657 AATTGGTGCTCCAGATATAGCATTCCCTCGACTTAATAATTTAAGTTTTTGATTATTAATTCCATCTTTATATTTATTAATTATAAGTAGTTTATTAGATATAGG.
TeKe14A/1-657 AATTGGTGCTCCAGATATAGCATTCCCTCGACTTAATAATTTAAGTTTTTGATTATTAATTCCATCTTTATATTTATTAATTATAAGTAGTTTATTAGATATAGG.
TeKe15A/1-657 AATTGGTGCTCCAGATATAGCATTCCCTCGACTTAATAATTTAAGTTTTTGATTATTAATTCCATCTTTATATTTATTAATTATAAGTAGTTTATTAGATATAGG.
TeKe1A/1-657 AATTGGTGCTCCAGATATAGCATTCCCTCGACTTAATAATTTAAGTTTTTGATTATTAATTCCATCTTTATATTTATTAATTATAAGTAGTTTATTAGATATAGG.
TeKe3B/1-657 AATTGGTGCTCCAGATATAGCATTCCCTCGACTTAATAATTTAAGTTTTTGATTATTAATTCCATCTTTATATTTATTAATTATAAGTAGTTTATTAGATATAGG.
TeKe4A/1-657 AATTGGTGCTCCAGATATAGCATTCCCTCGACTTAATAATTTAAGTTTTTGATTATTAATTCCATCTTTATATTTATTAATTATAAGTAGTTTATTAGATATAGG.
TeKe5A/1-657 AATTGGTGCTCCAGATATAGCATTCCCTCGACTTAATAATTTAAGTTTTTGATTATTAATTCCATCTTTATATTTATTAATTATAAGTAGTTTATTAGATATAGG.
TeKe7A/1-657 AATTGGTGCTCCAGATATAGCATTCCCTCGACTTAATAATTTAAGTTTTTGATTATTAATTCCATCTTTATATTTATTAATTATAAGTAGTTTATTAGATATAGG.
TeKe8A/1-657 AATTGGTGCTCCAGATATAGCATTCCCTCGACTTAATAATTTAAGTTTTTGATTATTAATTCCATCTTTATATTTATTAATTATAAGTAGTTTATTAGATATAGG.
TeKe9A/1-657 AATTGGTGCTCCAGATATAGCATTCCCTCGACTTAATAATTTAAGTTTTTGATTATTAATTCCATCTTTATATTTATTAATTATAAGTAGTTTATTAGATATAGG.
TeKe2A/1-657 AATTGGTGCTCCAGATATAGCATTCCCTCGACTTAATAATTTAAGTTTTTGATTATTAATTCCATCTTTATATTTATTAATTATAAGTAGTTTATTAGATATAGG.
TeTz2/1-657 AATTGGTGCTCCAGATATAGCATTCCCTCGACTTAATAATTTAAGTTTTTGATTATTAATTCCATCTTTATATTTATTAATTATAAGTAGTTTATTAGATATAGG.
Uganda_MT416549/36-692 AATTGGTGCTCCAGATATAGCATTCCCTCGACTTAATAATTTAAGTTTTTGATTATTAATTCCATCTTTATATTTATTAATTATAAGTAGTTTACTAGATATAGG.
HomaBay-252/1-657 AATTGGTGCTCCAGATATAGCATTCCCTCGACTTAATAATTTAAGTTTTTGATTATTAATTCCATCTTTATATTTATTAATTATAAGTAGTTTACTAGATATAGG.
HomaBay-254/1-657 AATTGGTGCTCCAGATATAGCATTCCCTCGACTTAATAATTTAAGTTTTTGATTATTAATTCCATCTTTATATTTATTAATTATAAGTAGTTTACTAGATATAGG.
Ethiopia_MT416551/36-692 AATTGGTGCTCCAGATATAGCATTCCCTCGACTTAATAATTTAAGTTTTTGATTATTAATTCCATCTTTATATTTATTAATTATAAGTAGTTTACTAGATATAGG.
Cameroon_MG989238/3091-3747 AATTGGTGCTCCAGATATAGCATTCCCTCGACTTAATAATTTAAGTTTTTGATTATTAATTCCATCTTTATATTTATTAATTATAAGTAGTTTACTAGATATAGG.
Moreno_178/1-657 AATTGGTGCTCCAGATATAGCATTTCCTCGACTTAATAATTTAAGTTTTTGATTATTAATTCCATCTTTATATTTATTAATTATAAGTAGTTTATTAGATATAGG.
AguaGarcia-95_MK285551/19-675 AATTGGTGCTCCAGATATAGCATTTCCTCGACTTAATAATTTAAGTTTTTGATTATTAATTCCATCTTTATATTTATTAATTATAAGTAGTTTATTAGATATAGG.
Aldan-72_MK285548/31-687 AATTGGTGCTCCAGATATAGCATTTCCTCGACTTAATAATTTAAGTTTTTGATTATTAATTCCATCTTTATATTTATTAATTATAAGTAGTTTATTAGATATAGG.
Aldan-91/19-675 AATTGGTGCTCCAGATATAGCATTTCCTCGACTTAATAATTTAAGTTTTTGATTATTAATTCCATCTTTATATTTATTAATTATAAGTAGTTTATTAGATATAGG.
Areeiro-Lis-231/19-675 AATTGGTGCTCCAGATATAGCATTTCCTCGACTTAATAATTTAAGTTTTTGATTATTAATTCCATCTTTATATTTATTAATTATAAGTAGTTTATTAGATATAGG.
Areeiro-77_MK285549/19-675 AATTGGTGCTCCAGATATAGCATTTCCTCGACTTAATAATTTAAGTTTTTGATTATTAATTCCATCTTTATATTTATTAATTATAAGTAGTTTATTAGATATAGG.
Areeiro-81/19-675 AATTGGTGCTCCAGATATAGCATTTCCTCGACTTAATAATTTAAGTTTTTGATTATTAATTCCATCTTTATATTTATTAATTATAAGTAGTTTATTAGATATAGG.
Barreiralva-228/1-657 AATTGGTGCTCCAGATATAGCATTTCCTCGACTTAATAATTTAAGTTTTTGATTATTAATTCCATCTTTATATTTATTAATTATAAGTAGTTTATTAGATATAGG.
Camacha-210/1-657 AATTGGTGCTCCAGATATAGCATTTCCTCGACTTAATAATTTAAGTTTTTGATTATTAATTCCATCTTTATATTTATTAATTATAAGTAGTTTATTAGATATAGG.
Farrobo-189/1-657 AATTGGTGCTCCAGATATAGCATTTCCTCGACTTAATAATTTAAGTTTTTGATTATTAATTCCATCTTTATATTTATTAATTATAAGTAGTTTATTAGATATAGG.
GranCanaria-11/1-657 AATTGGTGCTCCAGATATAGCATTTCCTCGACTTAATAATTTAAGTTTTTGATTATTAATTCCATCTTTATATTTATTAATTATAAGTAGTTTATTAGATATAGG.
GranCanaria-5_MK285553/1-657 AATTGGTGCTCCAGATATAGCATTTCCTCGACTTAATAATTTAAGTTTTTGATTATTAATTCCATCTTTATATTTATTAATTATAAGTAGTTTATTAGATATAGG.
GranCanaria-7/1-657 AATTGGTGCTCCAGATATAGCATTTCCTCGACTTAATAATTTAAGTTTTTGATTATTAATTCCATCTTTATATTTATTAATTATAAGTAGTTTATTAGATATAGG.
GranCanaria-8/1-657 AATTGGTGCTCCAGATATAGCATTTCCTCGACTTAATAATTTAAGTTTTTGATTATTAATTCCATCTTTATATTTATTAATTATAAGTAGTTTATTAGATATAGG.
GranCanaria-9/1-657 AATTGGTGCTCCAGATATAGCATTTCCTCGACTTAATAATTTAAGTTTTTGATTATTAATTCCATCTTTATATTTATTAATTATAAGTAGTTTATTAGATATAGG.
Orotava-66/31-687 AATTGGTGCTCCAGATATAGCATTTCCTCGACTTAATAATTTAAGTTTTTGATTATTAATTCCATCTTTATATTTATTAATTATAAGTAGTTTATTAGATATAGG.
Orotava-67/31-687 AATTGGTGCTCCAGATATAGCATTTCCTCGACTTAATAATTTAAGTTTTTGATTATTAATTCCATCTTTATATTTATTAATTATAAGTAGTTTATTAGATATAGG.
Orotava-68-MK285552/19-675 AATTGGTGCTCCAGATATAGCATTTCCTCGACTTAATAATTTAAGTTTTTGATTATTAATTCCATCTTTATATTTATTAATTATAAGTAGTTTATTAGATATAGG.
Paiao-236/1-657 AATTGGTGCTCCAGATATAGCATTTCCTCGACTTAATAATTTAAGTTTTTGATTATTAATTCCATCTTTATATTTATTAATTATAAGTAGTTTATTAGATATAGG.
Poiso-184/1-657 AATTGGTGCTCCAGATATAGCATTTCCTCGACTTAATAATTTAAGTTTTTGATTATTAATTCCATCTTTATATTTATTAATTATAAGTAGTTTATTAGATATAGG.
Portonovo-25_MK285550/1-657 AATTGGTGCTCCAGATATAGCATTTCCTCGACTTAATAATTTAAGTTTTTGATTATTAATTCCATCTTTATATTTATTAATTATAAGTAGTTTATTAGATATAGG.
Portonovo-30/1-657 AATTGGTGCTCCAGATATAGCATTTCCTCGACTTAATAATTTAAGTTTTTGATTATTAATTCCATCTTTATATTTATTAATTATAAGTAGTTTATTAGATATAGG.
Pretoria-53/31-687 AATTGGTGCTCCAGATATAGCATTTCCTCGACTTAATAATTTAAGTTTTTGATTATTAATTCCATCTTTATATTTATTAATTATAAGTAGTTTATTAGATATAGG.
Pretoria-54_MK285559/31-687 AATTGGTGCTCCAGATATAGCATTTCCTCGACTTAATAATTTAAGTTTTTGATTATTAATTCCATCTTTATATTTATTAATTATAAGTAGTTTATTAGATATAGG.
Pretoria-57/31-687 AATTGGTGCTCCAGATATAGCATTTCCTCGACTTAATAATTTAAGTTTTTGATTATTAATTCCATCTTTATATTTATTAATTATAAGTAGTTTATTAGATATAGG.
Pretoria-58/31-687 AATTGGTGCTCCAGATATAGCATTTCCTCGACTTAATAATTTAAGTTTTTGATTATTAATTCCATCTTTATATTTATTAATTATAAGTAGTTTATTAGATATAGG.
Pretoria-99/31-687 AATTGGTGCTCCAGATATAGCATTTCCTCGACTTAATAATTTAAGTTTTTGATTATTAATTCCATCTTTATATTTATTAATTATAAGTAGTTTATTAGATATAGG.
Quebradas-59/31-687 AATTGGTGCTCCAGATATAGCATTTCCTCGACTTAATAATTTAAGTTTTTGATTATTAATTCCATCTTTATATTTATTAATTATAAGTAGTTTATTAGATATAGG.
Madeira-60_MK285558/31-687 AATTGGTGCTCCAGATATAGCATTTCCTCGACTTAATAATTTAAGTTTTTGATTATTAATTCCATCTTTATATTTATTAATTATAAGTAGTTTATTAGATATAGG.
Quebradas-61/31-687 AATTGGTGCTCCAGATATAGCATTTCCTCGACTTAATAATTTAAGTTTTTGATTATTAATTCCATCTTTATATTTATTAATTATAAGTAGTTTATTAGATATAGG.
Ribamar-192/1-657 AATTGGTGCTCCAGATATAGCATTTCCTCGACTTAATAATTTAAGTTTTTGATTATTAATTCCATCTTTATATTTATTAATTATAAGTAGTTTATTAGATATAGG.
SaoVicente-198/1-657 AATTGGTGCTCCAGATATAGCATTTCCTCGACTTAATAATTTAAGTTTTTGATTATTAATTCCATCTTTATATTTATTAATTATAAGTAGTTTATTAGATATAGG.
Sobreda-244/1-657 AATTGGTGCTCCAGATATAGCATTTCCTCGACTTAATAATTTAAGTTTTTGATTATTAATTCCATCTTTATATTTATTAATTATAAGTAGTTTATTAGATATAGG.
TeSA1/1-657 AATTGGTGCTCCAGATATAGCATTTCCTCGACTTAATAATTTAAGTTTTTGATTATTAATTCCATCTTTATATTTATTAATTATAAGTAGTTTATTAGATATAGG.
TeSA7/1-657 AATTGGTGCTCCAGATATAGCATTTCCTCGACTTAATAATTTAAGTTTTTGATTATTAATTCCATCTTTATATTTATTAATTATAAGTAGTTTATTAGATATAGG.
Vairao-207/1-657 AATTGGTGCTCCAGATATAGCATTTCCTCGACTTAATAATTTAAGTTTTTGATTATTAATTCCATCTTTATATTTATTAATTATAAGTAGTTTATTAGATATAGG.
Nelspruit-34_MK285554/27-683 AATTGGTGCTCCAGATATAGCATTTCCTCGACTTAATAATTTAAGTTTTTGATTATTAATTCCATCTTTATATTTATTAATTATAAGTAGTTTATTAGATATAGG.
Nelspruit-35/31-687 AATTGGTGCTCCAGATATAGCATTTCCTCGACTTAATAATTTAAGTTTTTGATTATTAATTCCATCTTTATATTTATTAATTATAAGTAGTTTATTAGATATAGG.
Nelspruit-38_MK285555/27-683 AATTGGTGCTCCAGATATAGCATTTCCTCGACTTAATAATTTAAGTTTTTGATTATTAATTCCATCTTTATATTTATTAATTATAAGTAGTTTATTAGATATAGG.
Nelspruit-40/27-683 AATTGGTGCTCCAGATATAGCATTTCCTCGACTTAATAATTTAAGTTTTTGATTATTAATTCCATCTTTATATTTATTAATTATAAGTAGTTTATTAGATATAGG.
Nelspruit-41/21-683 AATTGGTGCTCCAGATATAGCATTTCCTCGACTTAATAATTTAAGTTTTTGATTATTAATTCCATCTTTATATTTATTAATTATAAGTAGTTTATTAGATATAGG.
Nelspruit-42/27-683 AATTGGTGCTCCAGATATAGCATTTCCTCGACTTAATAATTTAAGTTTTTGATTATTAATTCCATCTTTATATTTATTAATTATAAGTAGTTTATTAGATATAGG.
Pretoria-100_MK285560/1-657 AATTGGTGCTCCAGATATAGCATTTCCTCGACTTAATAATTTAAGTTTTTGATTATTAATTCCATCTTTATATTTATTAATTATAAGTAGTTTATTAGATATAGG.
SouthAfrica_MT416550/36-692 AATTGGTGCTCCAGATATAGCATTTCCTCGACTTAATAATTTAAGTTTTTGATTATTAATTCCATCTTTATATTTATTAATTATAAGTAGTTTATTAGATATAGG.
Tzaneen-43/27-683 AATTGGTGCTCCAGATATAGCATTTCCTCGACTTAATAATTTAAGTTTTTGATTATTAATTCCATCTTTATATTTATTAATTATAAGTAGTTTATTAGATATAGG.
Tzaneen-46/27-683 AATTGGTGCTCCAGATATAGCATTTCCTCGACTTAATAATTTAAGTTTTTGATTATTAATTCCATCTTTATATTTATTAATTATAAGTAGTTTATTAGATATAGG.
Tzaneen-47/27-683 AATTGGTGCTCCAGATATAGCATTTCCTCGACTTAATAATTTAAGTTTTTGATTATTAATTCCATCTTTATATTTATTAATTATAAGTAGTTTATTAGATATAGG.
Tzaneen-48/27-683 AATTGGTGCTCCAGATATAGCATTTCCTCGACTTAATAATTTAAGTTTTTGATTATTAATTCCATCTTTATATTTATTAATTATAAGTAGTTTATTAGATATAGG.
Tzaneen-50/27-683 AATTGGTGCTCCAGATATAGCATTTCCTCGACTTAATAATTTAAGTTTTTGATTATTAATTCCATCTTTATATTTATTAATTATAAGTAGTTTATTAGATATAGG.
Tzaneen-52_MK285557/27-683 AATTGGTGCTCCAGATATAGCATTTCCTCGACTTAATAATTTAAGTTTTTGATTATTAATTCCATCTTTATATTTATTAATTATAAGTAGTTTATTAGATATAGG.
Tzaneen-44/27-683 AATTGGTGCTCCAGATATAGCATTTCCTCGACTTAATAATTTAAGTTTTTGATTATTAATTCCATCTTTATATTTATTAATTATAAGTAGTTTATTAGATATAGG.
Tzaneen-45_MK285556/27-683 AATTGGTGCTCCAGATATAGCATTTCCTCGACTTAATAATTTAAGTTTTTGATTATTAATTCCATCTTTATATTTATTAATTATAAGTAGTTTATTAGATATAGG.
Tzaneen-49/27-683 AATTGGTGCTCCAGATATAGCATTTCCTCGACTTAATAATTTAAGTTTTTGATTATTAATTCCATCTTTATATTTATTAATTATAAGTAGTTTATTAGATATAGG.
TeSA10/1-657 AATTGGTGCTCCAGATATAGCATTTCCTCGACTTAATAATTTAAGTTTTTGATTATTAATTCCATCTTTATATTTATTAATTATAAGTAGTTTATTAGATATAGG.
TeSA3/1-657 AATTGGTGCTCCAGATATAGCATTTCCTCGACTTAATAATTTAAGTTTTTGATTATTAATTCCATCTTTATATTTATTAATTATAAGTAGTTTATTAGATATAGG.
TeSA5/1-657 AATTGGTGCTCCAGATATAGCATTTCCTCGACTTAATAATTTAAGTTTTTGATTATTAATTCCATCTTTATATTTATTAATTATAAGTAGTTTATTAGATATAGG.
TeSA6/1-657 AATTGGTGCTCCAGATATAGCATTTCCTCGACTTAATAATTTAAGTTTTTGATTATTAATTCCATCTTTATATTTATTAATTATAAGTAGTTTATTAGATATAGG.

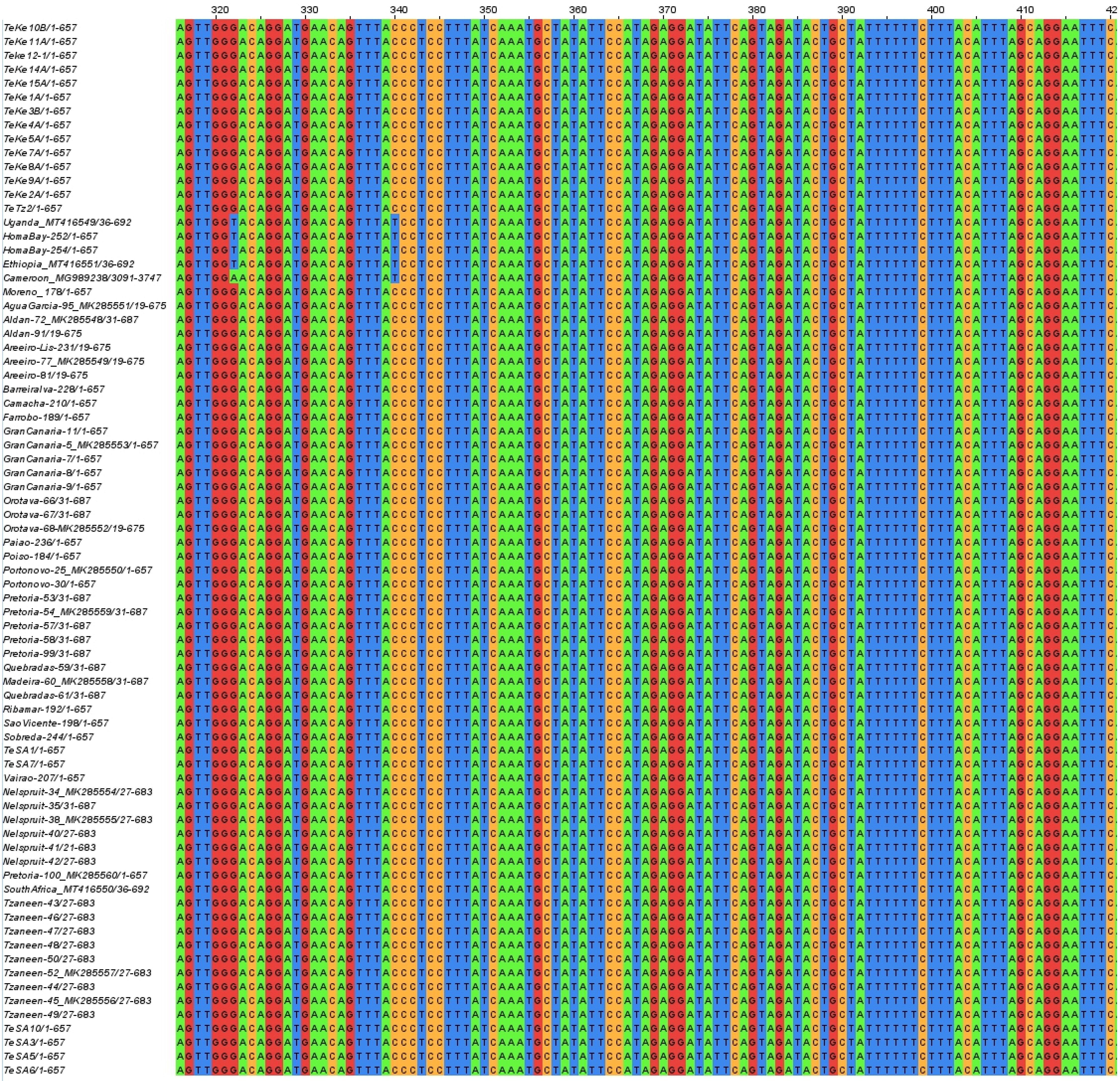
320
330
340
350
360
370
380
390
400
410
42
TeKe10B/1-657
TeKe11A/1-657
Teke12-1/1-657
TeKe14A/1-657
TeKe15A/1-657
TeKe1A/1-657
TeKe3B/1-657
TeKe4A/1-657
TeKe5A/1-657
TeKe7A/1-657
TeKe8A/1-657
TeKe9A/1-657
TeKe2A/1-657
TeTz2/1-657
Uganda_MT416549/36-692
HomaBay-252/1-657
HomaBay-254/1-657
Ethiopia_MT416551/36-692
Cameroon_MG989238/3091-3747
Moreno_178/1-657
AguaGarcia-95_MK285551/19-675
Aldan-72_MK285548/31-687
Aldan-91/19-675
Areeiro-Lis-231/19-675
Areeiro-77_MK285549/19-675
Areeiro-81/19-675
Barreiralva-228/1-657
Camacha-210/1-657
Farrobo-189/1-657
GranCanaria-11/1-657
GranCanaria-5_MK285553/1-657
GranCanaria-7/1-657
GranCanaria-8/1-657
GranCanaria-9/1-657
Orotava-66/31-687
Orotava-67/31-687
Orotava-68-MK285552/19-675
Paiao-236/1-657
Poiso-184/1-657
Portonovo-25_MK285550/1-657
Portonovo-30/1-657
Pretoria-53/31-687
Pretoria-54_MK285559/31-687
Pretoria-57/31-687
Pretoria-58/31-687
Pretoria-99/31-687
Quebradas-59/31-687
Madeira-60_MK285558/31-687
Quebradas-61/31-687
Ribamar-192/1-657
SaoVicente-198/1-657
Sobreda-244/1-657
TeSA1/1-657
TeSA7/1-657
Vairao-207/1-657
Nelspruit-34_MK285554/27-683
Nelspruit-35/31-687
Nelspruit-38_MK285555/27-683
Nelspruit-40/27-683
Nelspruit-41/21-683
Nelspruit-42/27-683
Pretoria-100_MK285560/1-657
SouthAfrica_MT416550/36-692
Tzaneen-43/27-683
Tzaneen-46/27-683
Tzaneen-47/27-683
Tzaneen-48/27-683
Tzaneen-50/27-683
Tzaneen-52_MK285557/27-683
Tzaneen-44/27-683
Tzaneen-45_MK285556/27-683
Tzaneen-49/27-683
TeSA10/1-657
TeSA3/1-657
TeSA5/1-657
TeSA6/1-657

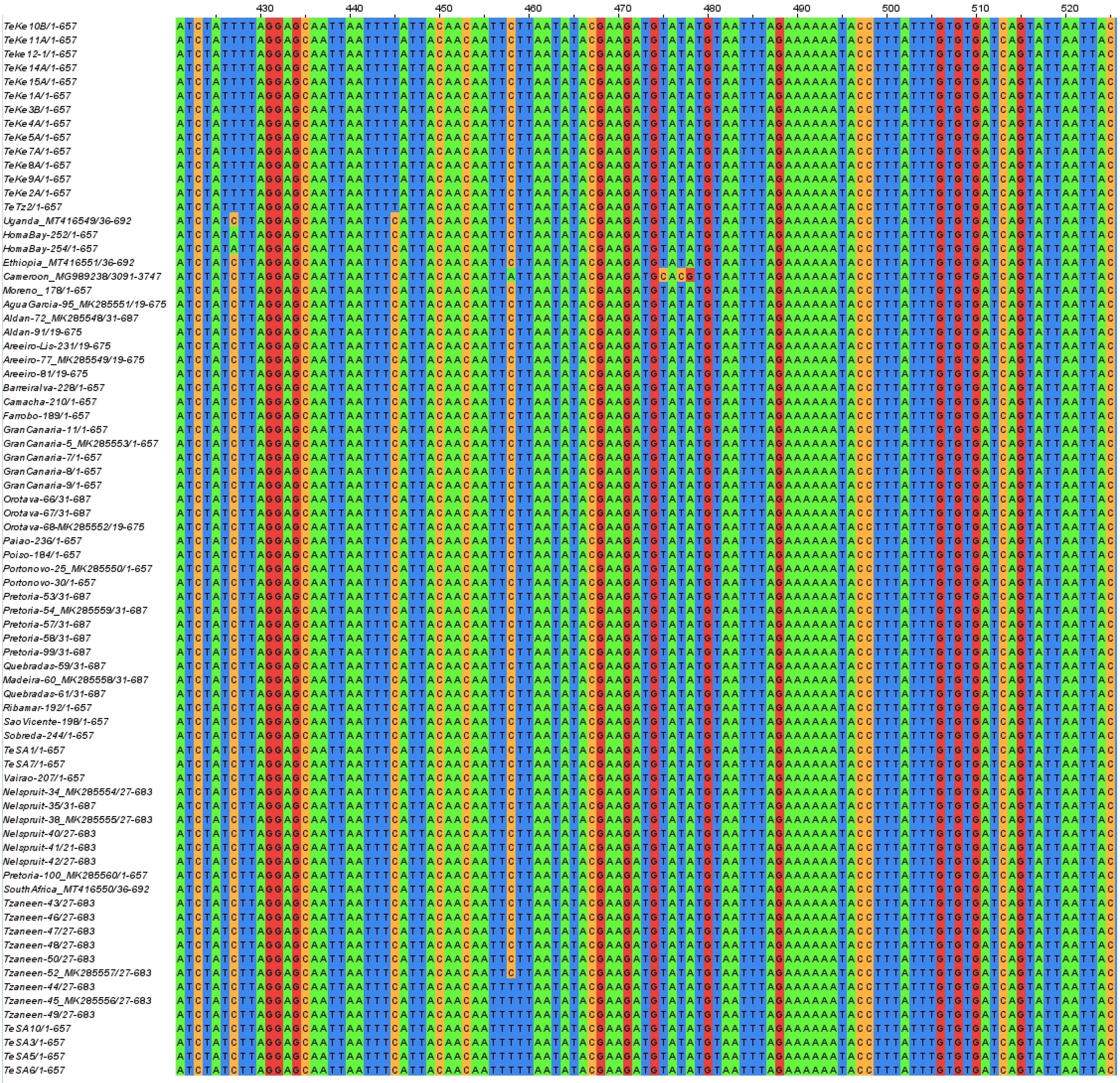
430
440
450
460
470
480
490
500
510
520
TeKe10B/1-657
TeKe11A/1-657
Teke12-1/1-657
TeKe14A/1-657
TeKe15A/1-657
TeKe1A/1-657
TeKe3B/1-657
TeKe4A/1-657
TeKe5A/1-657
TeKe7A/1-657
TeKe8A/1-657
TeKe9A/1-657
TeKe2A/1-657
TeTz2/1-657
Uganda_MT416549/36-692
HomaBay-252/1-657
HomaBay-254/1-657
Ethiopia_MT416551/36-692
Cameroon_MG989238/3091-3747
Moreno_178/1-657
AguaGarcia-95_MK285551/19-675
Aldan-72_MK285548/31-687
Aldan-91/19-675
Areeiro-Lis-231/19-675
Areeiro-77_MK285549/19-675
Areeiro-81/19-675
Barreiralva-228/1-657
Camacha-210/1-657
Farrobo-189/1-657
GranCanaria-11/1-657
GranCanaria-5_MK285553/1-657
GranCanaria-7/1-657
GranCanaria-8/1-657
GranCanaria-9/1-657
Orotava-66/31-687
Orotava-67/31-687
Orotava-68-MK285552/19-675
Paiao-236/1-657
Poiso-184/1-657
Portonovo-25_MK285550/1-657
Portonovo-30/1-657
Pretoria-53/31-687
Pretoria-54_MK285559/31-687
Pretoria-57/31-687
Pretoria-58/31-687
Pretoria-99/31-687
Quebradas-59/31-687
Madeira-60_MK285558/31-687
Quebradas-61/31-687
Ribamar-192/1-657
SaoVicente-198/1-657
Sobreda-244/1-657
TeSA1/1-657
TeSA7/1-657
Vairao-207/1-657
Nelspruit-34_MK285554/27-683
Nelspruit-35/31-687
Nelspruit-38_MK285555/27-683
Nelspruit-40/27-683
Nelspruit-41/21-683
Nelspruit-42/27-683
Pretoria-100_MK285560/1-657
SouthAfrica_MT416550/36-692
Tzaneen-43/27-683
Tzaneen-46/27-683
Tzaneen-47/27-683
Tzaneen-48/27-683
Tzaneen-50/27-683
Tzaneen-52_MK285557/27-683
Tzaneen-44/27-683
Tzaneen-45_MK285556/27-683
Tzaneen-49/27-683
TeSA10/1-657
TeSA3/1-657
TeSA5/1-657
TeSA6/1-657

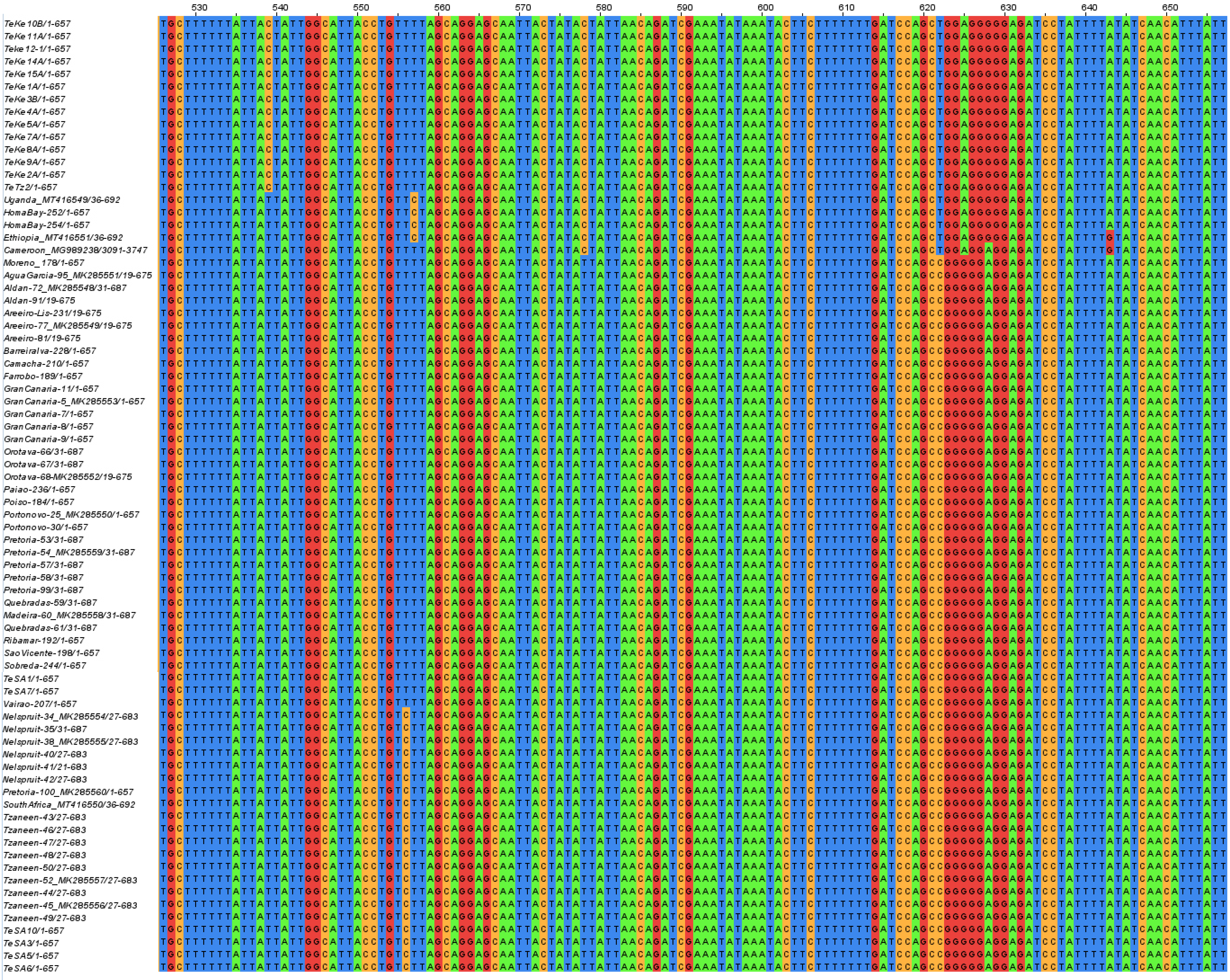
530
540
550
560
570
580
590
600
610
620
630
640
650
TeKe10B/1-657
TeKe11A/1-657
Teke12-1/1-657
TeKe14A/1-657
TeKe15A/1-657
TeKe1A/1-657
TeKe3B/1-657
TeKe4A/1-657
TeKe5A/1-657
TeKe7A/1-657
TeKe8A/1-657
TeKe9A/1-657
TeKe2A/1-657
TeTz2/1-657
Uganda_MT416549/36-692
HomaBay-252/1-657
HomaBay-254/1-657
Ethiopia_MT416551/36-692
Cameroon_MG989238/3091-3747
Moreno_178/1-657
AguaGarcia-95_MK285551/19-675
Aldan-72_MK285548/31-687
Aldan-91/19-675
Areeiro-Lis-231/19-675
Areeiro-77_MK285549/19-675
Areeiro-81/19-675
Barreiralva-228/1-657
Camacha-210/1-657
Farrobo-189/1-657
GranCanaria-11/1-657
GranCanaria-5_MK285553/1-657
GranCanaria-7/1-657
GranCanaria-8/1-657
GranCanaria-9/1-657
Orotava-66/31-687
Orotava-67/31-687
Orotava-68-MK285552/19-675
Paiao-236/1-657
Poiso-184/1-657
Portonovo-25_MK285550/1-657
Portonovo-30/1-657
Pretoria-53/31-687
Pretoria-54_MK285559/31-687
Pretoria-57/31-687
Pretoria-58/31-687
Pretoria-99/31-687
Quebradas-59/31-687
Madeira-60_MK285558/31-687
Quebradas-61/31-687
Ribamar-192/1-657
SaoVicente-198/1-657
Sobreda-244/1-657
TeSA1/1-657
TeSA7/1-657
Vairao-207/1-657
Nelspruit-34_MK285554/27-683
Nelspruit-35/31-687
Nelspruit-38_MK285555/27-683
Nelspruit-40/27-683
Nelspruit-41/21-683
Nelspruit-42/27-683
Pretoria-100_MK285560/1-657
SouthAfrica_MT416550/36-692
Tzaneen-43/27-683
Tzaneen-46/27-683
Tzaneen-47/27-683
Tzaneen-48/27-683
Tzaneen-50/27-683
Tzaneen-52_MK285557/27-683
Tzaneen-44/27-683
Tzaneen-45_MK285556/27-683
Tzaneen-49/27-683
TeSA10/1-657
TeSA3/1-657
TeSA5/1-657
TeSA6/1-657
