# Supplementary material for: Insights into the origin of the invasive populations of Trioza erytreae in Europe using microsatellite markers and mtDNA barcoding approaches
Source: Sci Rep. 2021 Sep 20;11:18651. doi: 10.1038/s41598-021-97824-0 (PMC8452619; doi:10.1038/s41598-021-97824-0)
Supplement: Supplementary file 5 — Supplementary Information 5. [file 41598_2021_97824_MOESM5_ESM.docx]

**Supplementary Data Legends**

**Supplementary Data 1.** Genomic SSR distribution.

**Supplementary Data 2.** Membership coefficient (*Q*) for individuals at each cluster

**Supplementary Data 3.** ClustalW alignment of 39 COI barcode DNA fragments generated from this study and 37 accessions retrieved from the GenBank. Coordinates are given with respect to the original nucleotide sequences, except for the accessions MG989238, MT416549, MT416550, and MT416551 for which the coordinates are given with respect to the whole mitochondrial genome sequence of *Trioza erytreae*. For sequence alignment, a 657 bp fragment, from positions +6 to +719 with respect to the start codon of the COI coding region, was used.

**Supplementary Data 4.** Percentage of nucleotide sequence identity of the 657 bp COI barcode fragment sequenced from *Trioza erytreae* individuals.
